# Type I IFN protects cancer cells from CD8^+^ T cell–mediated cytotoxicity after radiation

**DOI:** 10.1172/JCI127458

**Published:** 2019-09-04

**Authors:** Jianzhou Chen, Yunhong Cao, Bostjan Markelc, Jakob Kaeppler, Jenny A.F. Vermeer, Ruth J. Muschel

**Affiliations:** 1 CRUK/MRC Oxford Institute for Radiation Oncology, Department of Oncology, University of Oxford, Oxford, United Kingdom.; 2 Department of Radiation Oncology, Cancer Hospital of Shantou University Medical College, Shantou, China.

**Keywords:** Immunology, Oncology, Cancer, Cellular immune response, Radiation therapy

## Abstract

Treatment of tumors with ionizing radiation stimulates an antitumor immune response partly dependent on induction of IFNs. These IFNs directly enhance dendritic cell and CD8^+^ T cell activity. Here we show that resistance to an effective antitumor immune response is also a result of IFN signaling in a different cellular compartment of the tumor, the cancer cells themselves. We abolished type I IFN signaling in cancer cells by genetic elimination of its receptor, IFNAR1. Pronounced immune responses were provoked after ionizing radiation of tumors from 4 mouse cancer cell lines with *Ifnar1* knockout. This enhanced response depended on CD8^+^ T cells and was mediated by enhanced susceptibility to T cell–mediated killing. Induction of *Serpinb9* proved to be the mechanism underlying control of susceptibility to T cell killing after radiation. *Ifnar1*-deficient tumors had an augmented response to anti–PD-L1 immunotherapy with or without radiation. We conclude that type I IFN can protect cancer cells from T cell–mediated cytotoxicity through regulation of *Serpinb9*. This result helps explain why radiation of tumors can stimulate antitumor immunity yet also result in resistance. It further suggests potential targets for intervention to improve therapy and to predict responses.

## Introduction

The conflicting effects of type I IFN signaling in the immune response are well recognized. IFNs, both type I IFN and IFN-γ (IFNG), facilitate DC and CD8^+^ T cell activity and T cell cross-priming. However, IFN can also lead to immunosuppression (1, 2). The role of IFNs in cancer and in response to therapy is similarly complex. In some studies abrogation of IFN signaling in the host or in cancer cells was shown to diminish localized or systemic immune responses after radiation therapy with or without immune checkpoint blockade (3–5). However, persistent IFN signaling led to resistance to immune checkpoint therapy in other experimental models (6).

Radiotherapy is one of the main treatment modalities for cancer. It is now well accepted that host immunity has a pivotal impact on the therapeutic outcomes of radiotherapy in animal models (7–9). In humans addition of an anti–programmed death ligand 1 (anti–PD-L1) Ab to standard chemoradiotherapy for locally advanced non–small cell lung cancer led to substantially better outcomes, although more than 50% of patients still experienced subsequent disease progression (10). Very infrequently localized radiotherapy to one lesion leads to regression of other nonirradiated lesions. This is termed the abscopal effect, which potentially can be enhanced with the addition of immunotherapy (9). Ionizing radiation (IR) boosts the antitumor immune response through induction of type I and III IFNs and immune cell death, leading to the release of damage-associated molecular patterns (DAMPs) (2, 5, 11, 12). IR induces DNA strand breakage, which can lead to formation of micronuclei (13, 14). Release of the contents of these micronuclei including ssDNA triggers the STING/TBK1 pathway, resulting in induction of IFNs. Although a low level of DNA damage is maintained in many cancer cells, leading to chronic activation of this pathway, the DNA damage induced by the doses of IR used in therapy greatly exceeds this baseline level and should trigger much higher levels of type I and perhaps type III IFNs (5, 12).

IFNs affect the efficacy of radiation therapy in murine models. Type I IFN in particular is a key factor in a radiation-induced immune response (3, 4). Interestingly, activation of type I IFN signaling in tumors can exert both beneficial and detrimental effects on the tumor response to radiotherapy. These effects often are mediated by immune responses. Genetic elimination of type I IFN receptors in the murine host or systemic blockade of type I IFN signaling via Ab abrogates the antitumor immune response after irradiation (3, 4). IR stimulates immunity through IFN-mediated induction of MHC class I on cancer cells (15), activates DCs through IFN signaling (3, 4, 16, 17), releases DAMPs (3, 4), increases the T cell repertoire (18–20), attracts effector T cells by induction of CXCL16 in cancer cells (21), and helps maintain stable interactions between tumor cells and T cells by upregulation of RAE-1 (22) — all stimulatory for antitumor immunity. Conversely, radiation and IFN can lead to resistance to the immune response. There is a growing body of evidence indicating that activation of type I IFN signaling in tumors correlates with worse outcomes in patients and with their resistance to therapies, including radiotherapy (23–26). In some experimental systems, radiation results in resistance to therapy due to expression of PD-L1, which could be mitigated by administration of an Ab blocking PD-L1 (27, 28). In addition, STING activation can lead to enhanced myeloid-derived suppressor cell infiltration, although this was not directly shown to be IFN based (29). Finally IFN can mediate resistance in the face of immune checkpoint inhibition with anti–CTLA-4 or anti–PD-L1 combined with radiation (6). Taken together, these series of results suggest that type I IFN signaling is likely to have opposing effects in tumors.

Some of the differing effects of IFN may reflect actions on different cell types in the tumor microenvironment. Since all cells express type I IFN receptors, its effects will depend on the aggregate of the response of each cell in the tumor. IFN signaling in DCs and T cells augments the immune response to tumors after radiation (1, 3, 4, 16, 17). The effects of type I IFN signaling in the cancer cell are less well defined, especially in the context of radiotherapy. Accordingly, we investigated the effects of type I IFN signaling in cancer cells by genetic elimination of *Ifnar1*. Cancer cells lacking *Ifnar1* gave rise to tumors in murine models, which grew similarly to WT cells yet had a greatly enhanced response to IR. This response to cancer cells lacking *Ifnar1* depended upon CD8^+^ T cell–based immunity and was mediated by enhanced susceptibility to CD8^+^ T cell–mediated killing. We found that downregulation of *Serpinb9*, an IFN-inducible gene in *Ifnar1*-deficient cells, greatly reduced resistance to T cell killing after radiation.

## Results

### Abrogation of type I IFN signaling in cancer cells enhanced tumor response to IR.

**Figure 1:**
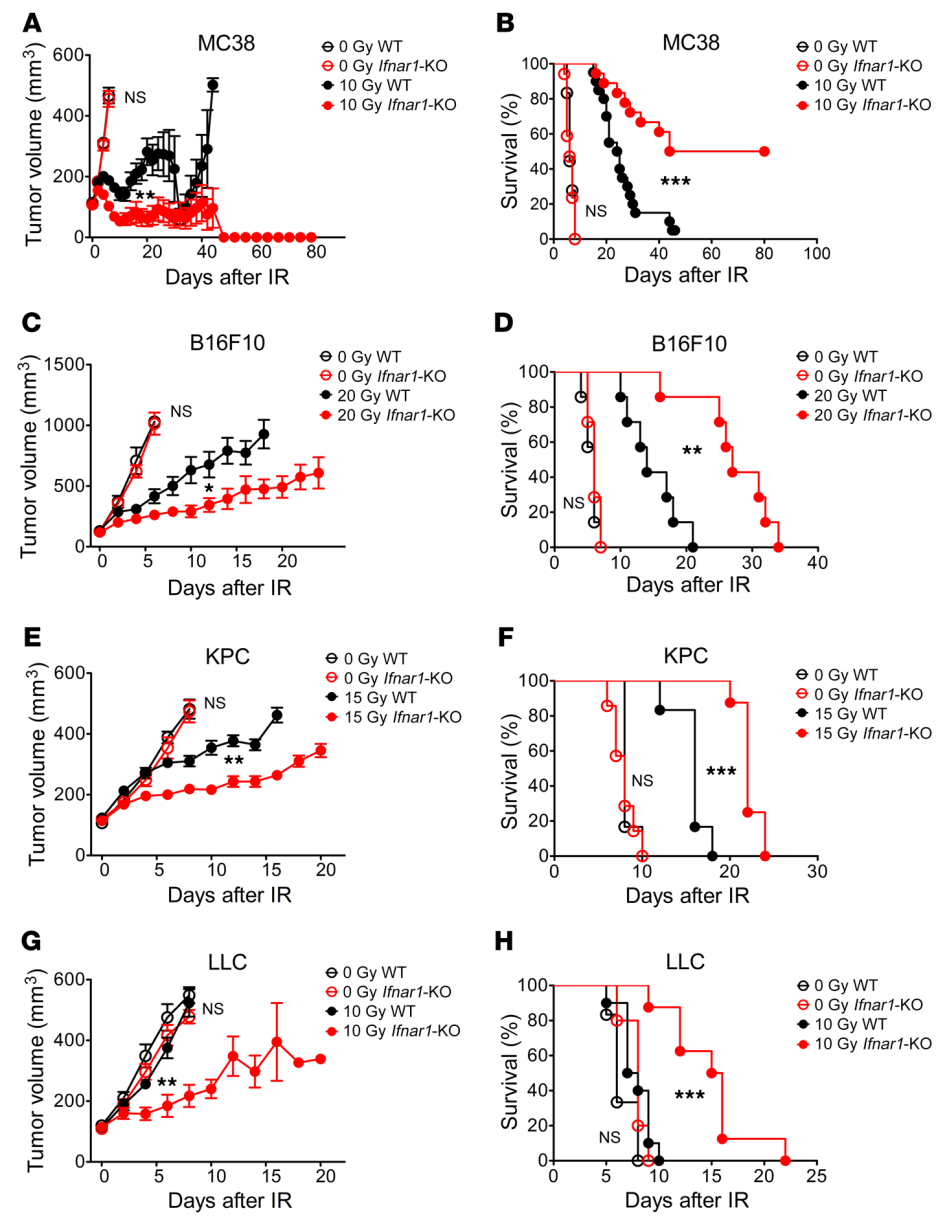
*Ifnar1* KO in cancer cells enhances tumor response to IR. Tumors in C57BL/6 mice derived from the indicated cancer cell lines with or without *Ifnar1* KO, including MC38 (**A** and **B**), B16F10 (**C** and **D**), KPC (**E** and **F**), and LLC (**G** and **H**) cells, received 0 Gy or the indicated single doses of IR. (**A**, **C**, **E**, and **G**) Tumor volume. Note that once mice had been culled due to reaching the ethically acceptable limit for tumor volume, the tumors from those mice no longer were included in the mean tumor volume calculation. (**B**, **D**, **F**, and **H**) Kaplan-Meier survival curves from the same experiment. *n* = 7–18 in control groups and 8–20 in irradiation groups. Error bars represent mean ± SEM. Comparison of 2 means was performed by the Mann-Whitney *U* test. Survival comparison between groups were performed using log-rank test (NS: *P* ≥ 0.05, *P < 0.05, **P < 0.01, ***P < 0.001).

To investigate the effects of type I IFN signaling in cancer cells, we prevented this signaling in 4 murine cancer cell lines: MC38 (colorectal carcinoma), B16F10 (melanoma), KPC (pancreatic cancer), and LLC (lung carcinoma). These cell lines vary in provoking a readily detectable immune response with infiltrating T cells (MC38), to intermediate responses (B16F10 and LLC), to those excluding T cells (KPC). IFNAR, the receptor for type I IFNs, is composed of 2 subunits, IFNAR1 and -2. To genetically abrogate its signaling, we used CRISPR/Cas9 methodology to inactivate *Ifnar1*. The *Ifnar1*-KO cell lines failed to respond to exogenous type I IFN by induction of the IFN-stimulated genes *Ifit1* and *Ifi44* (Supplemental Figure 1, A and B; supplemental material available online with this article; https://doi.org/10.1172/JCI127458DS1). There was no difference in growth in culture between *Ifnar1*-KO and parental B16F16, KPC, and LLC cells, while *Ifnar1*-KO MC38 cells grew faster in tissue culture than their WT counterpart MC38 cells (Supplemental Figure 1, C–F). In syngeneic C57BL/6 mice, tumors derived from the parental cells and the *Ifnar1*-KO cell lines grew at comparable rates without treatment (Figure 1).

We next examined the effect of IR. Clonogenic survival after radiation in tissue culture was equivalent for each *Ifnar1*-KO and parental cell line pair (Supplemental Figure 1, G–J). The extent of cellular apoptosis and cell cycle delay after radiation was equivalent for WT and *Ifnar1*-KO MC38 cells (Supplemental Figure 1, K and L). In contrast, substantial differences were found following IR of tumors. IR doses were chosen based on the radioresistance of each tumor model, with 10 Gy for MC38 and LLC, 20 Gy for B16F10, and 15 Gy for KPC. Administration of a single dose of 10 Gy IR led to a transient growth delay in WT MC38 tumors, with regression followed by recurrence. In comparison, tumors derived from *Ifnar1*-KO MC38 cells substantially regressed following radiation, and strikingly, 50% of mice remained tumor free (Figure 1A; growth of individual tumors is shown in Supplemental Figure 2). Kaplan-Meier survival analysis of these data revealed comparable differences (Figure 1B; median survival, 44 vs. 24 days, *P* < 0.001). In the 3 other models, the *Ifnar1*-KO tumors displayed significantly greater growth delay after IR compared with WT tumors, but without cure (Figure 1, C–H). Overall, genetic abrogation of type I IFN signaling in cancer cells enhanced the tumor response to IR.

### The enhanced response of Ifnar1-KO tumors to IR is mediated by CD8^+^ T cells.

**Figure 2:**
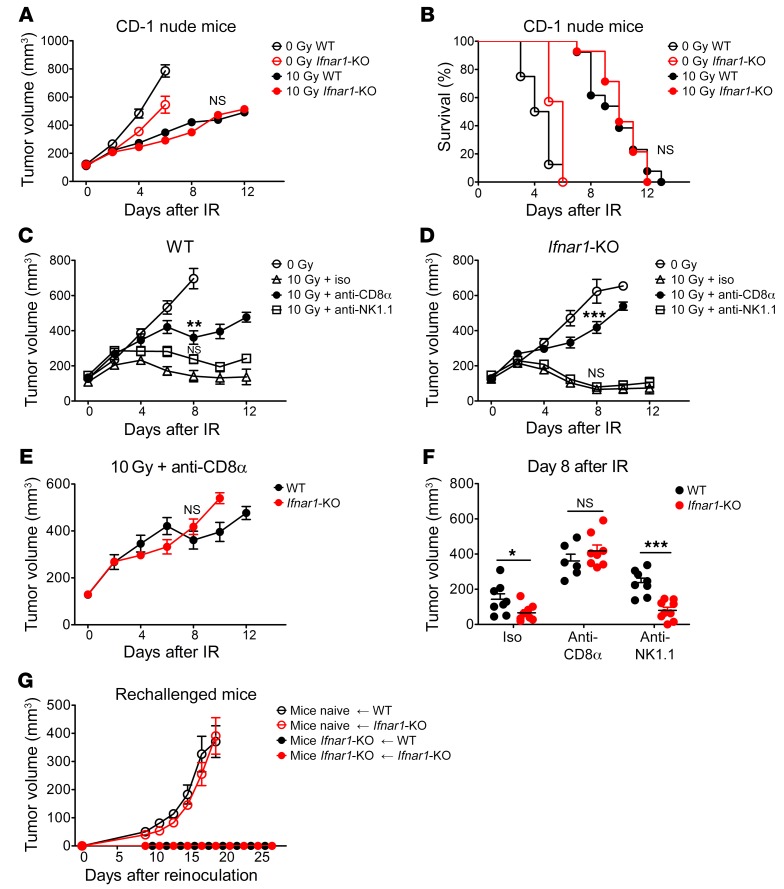
The enhanced response of *Ifnar1-*KO tumors to IR is mediated by CD8^+^ T cell immunity. MC38 tumors (WT and *Ifnar1*-KO) grown in CD-1 nude mice were treated with 0 Gy (*n* = 7–8) or 10 Gy (*n* = 13–14) IR. (**A** and **B**) Tumor volumes and mouse survival were assessed and summarized. C57BL/6 mice bearing subcutaneous WT (**C**) or *Ifnar1*-KO (**D**) MC38 tumors were subjected to the following treatments: 0 Gy IR; 10 Gy IR on day 0 plus isotype control Abs (10 Gy + iso); 10 Gy IR plus anti-CD8α Ab; and 10 Gy IR plus anti-NK1.1 Ab. Abs were administered on days –1, 2, 5, 8, and 11. *n* = 8–10. WT (**C**) or *Ifnar1*-KO (**D**) tumors with either CD8^+^ T cells or NK cells depleted were compared with tumors receiving isotype control Abs. (**E**) Growth of WT and *Ifnar1*-KO MC38 tumors following 10 Gy IR with CD8^+^ T cell depletion. (**F**) Mean volume of tumors on day 8 after IR was compared in WT and *Ifnar1*-KO mice. C57BL/6 mice with completely regressed *Ifnar1*-KO MC38 tumors after IR were rechallenged with WT or *Ifnar1*-KO MC38 cell on the other flank. (**G**) Growth of tumors following reinoculation. *n* = 4–6. Data show mean ± SEM (**A**–**E** and **G**) and mean ± SD (**F**). Comparison of 2 means was performed by the unpaired Student’s *t* test when data were normally distributed, and the Mann-Whitney *U* test when they were not or their normality could not be evaluated. Comparison of means of more than 2 groups was performed by 1-way ANOVA with Tukey’s multiple-comparisons test (NS: *P* ≥ 0.05, *P < 0.05, **P < 0.01, ***P < 0.001).

To evaluate whether an immune response played a role in these responses to IR, we performed an analogous experiment with MC38 tumors in immune-incompetent CD-1 nude mice. The host immune system is known to make an important contribution to IR-induced tumor control in the MC38 model (4, 16, 30). WT MC38 tumors in CD-1 nude mice exhibited growth retardation following IR, although to a lesser extent than in immunocompetent C57BL/6 mice. No significant differences in tumor control or host survival were found between WT and *Ifnar1*-KO MC38 tumors in the immunocompromised mice after radiation (Figure 2, A and B), implicating the host immune system as a factor affecting the increased sensitivity of *Ifnar1*-KO tumors to IR.

Both CD8^+^ T cells and NK cells, the 2 main populations of antitumor effector cells, can mediate the elimination of cancer cells in a tumor. To determine the contribution of these populations to the enhanced response to radiation of *Ifnar1*-KO tumors, we evaluated the effect of radiation on the growth of MC38 tumors with depletion of either population. Consistent with the results with IR alone (Figure 1A), IR plus isotype control Ab treatment led to significant retardation of growth of both WT and *Ifnar1*-KO tumors. With CD8^+^ T cell depletion the growth delay in response to IR was significantly reduced in WT MC38 tumors, and it was almost completely abolished in *Ifnar1*-KO tumors (Figure 2, C and D; depletion validated in Supplemental Figure 3). More importantly, no significant differences in tumor radiation response were found between WT and *Ifnar1*-KO MC38 tumors in CD8^+^ T cell–depleted mice, suggesting that CD8^+^ T cells are the main effector cells (Figure 2, E and F). NK depletion did not have evident effects on *Ifnar1*-KO tumors after IR, though there was a minor but not significant reduction of tumor response in WT MC38 tumors (Figure 2, C and D). *Ifnar1*-KO tumors in mice with NK cell depletion still showed substantially greater response to IR than WT tumors (Figure 2F). In results similar to those obtained with the MC38 model, T cell depletion in mice bearing tumors substantially reduced the growth delay after IR in the WT and *Ifnar1*-KO B16F10 and KPC tumors (Supplemental Figure 3, E–H). Collectively, these results suggest that the enhanced response of *Ifnar1*-KO tumors to IR is predominantly mediated by CD8^+^ T cell immunity.

We now asked whether mice in which *Ifnar1*-KO MC38 tumors had regressed retained systemic antitumor immunity to challenge with either WT or *Ifnar1*-KO cancer cells. Notably, neither WT nor *Ifnar1*-KO MC38 cells grew in mice that had previously rejected *Ifnar1*-KO tumors (Figure 2G). These data indicate that rejection was a systemic response involving memory. They also suggest that rejection was not due to recognition of a novel antigen presented by the *Ifnar1*-KO cells.

### CD8^+^ T cells infiltrating Ifnar1-KO tumors did not have augmented cytotoxic capacity or reduced exhaustion.

**Figure 3:**
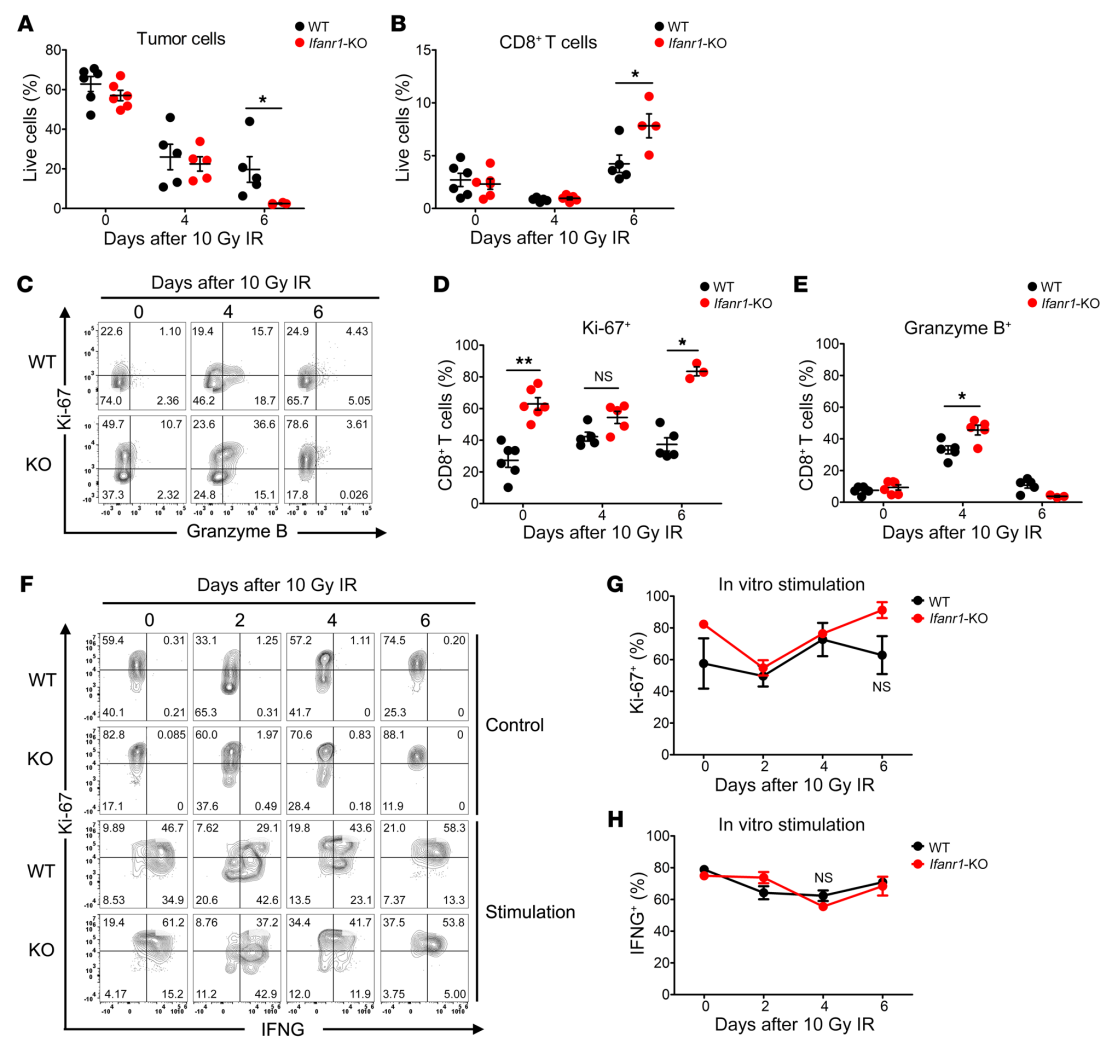
Characterization of infiltrating CD8^+^ T cells from *Ifnar1*-KO and WT MC38 tumors. WT or *Ifnar1*-KO MC38 tumors in C57BL/6 mice were subjected to 0 Gy or 10 Gy IR. On day 0, before IR, and days 4 and 6 after IR, tumors were harvested and disaggregated for cell type profiling using flow cytometry. (**A** and **B**) Percentages of tumor cells and CD8^+^ T cells among the total live cells. *n* = 5–6. (**C**) Representative flow cytometry plots characterizing gated CD8^+^ T cells, with Ki-67 on the *y* axis displayed against granzyme B on the *x* axis. (**D** and **E**) Percentages of Ki-67– and granzyme B–positive CD8^+^ T cells in WT or *Ifnar1*-KO tumors with or without IR. *n* = 5–6. CD8^+^ T cells isolated from WT or *Ifnar1*-KO MC38 tumors on days 0, 2, 4, and 6 following 10 Gy IR, were stimulated with PMA, ionomycin, and brefeldin A for 4 hours, and assessed by flow cytometry (representative plot shown in **F**). (**G** and **H**) Percentages of Ki-67– or IFNG-positive CD8^+^ T cells. *n* = 3–4. Data represent mean ± SD. Comparison of 2 means was performed by the Mann-Whitney *U* test (NS: *P* ≥ 0.05, *P < 0.05, **P < 0.01).

To understand how abrogation of type I IFN signaling in cancer cells led to better tumor control, we characterized CD8^+^ T cells in tumors from WT or *Ifnar1*-KO MC38 tumors after IR. The percentages of tumor cells and tumor-infiltrating CD8^+^ T cells were not markedly different at the time of radiation or 4 days later. By 6 days after radiation, the *Ifnar1*-KO MC38 tumors had increased infiltration of CD8^+^ T cells and a much smaller percentage of cancer cells, consistent with the decreasing volumes of *Ifnar1*-KO tumors at that time (Figure 1A and Figure 3, A and B). Although the percentages of CD8^+^ T cells infiltrating the different tumors were similar, the *Ifnar1*-KO tumors had elevated percentages with Ki-67 expression (Figure 3, C and D). Nonetheless, there was only a modest increase in the percentage of granzyme B–positive CD8^+^ T cells compared with those from WT tumors (Figure 3, C and E). At the later time, the percentage of granzyme B–positive CD8^+^ T cells had decreased in both groups. We evaluated the response of CD8^+^ T cells isolated from the tumors to stimulation (by PMA, ionomycin, and brefeldin A) in culture. CD8^+^ T cells from WT and *Ifnar1*-KO MC38 tumors isolated over a range of times after IR exhibited comparable IFNG induction in response to stimulation (Figure 3, F and H). There was a trend toward increased Ki-67 expression in CD8^+^ T cells from *Ifnar1*-KO tumors, but the differences were not statistically significant (Figure 3, F and G). Although granzyme B–positive CD8^+^ T cells were not as abundant in the other 3 models as in MC38 tumors, there was no significant increase in either granzyme B– or Ki-67–positive CD8^+^ T cells after IR in these *Ifnar1*-KO tumors (Supplemental Figure 4, A–D).

We next examined T cell exhaustion using 2 markers (PD-1 and T cell Ig and mucin domain 3 [TIM-3]) in CD8^+^ T cells, whose ligands (PD-L1 and LGALS9) are known to be expressed in some cancer cells from solid tumors. Most of the CD8^+^ T cells were PD-1 positive (75%–90%) in WT and *Ifnar1*-KO MC38 tumors, with a transient decrease on day 4 after IR in *Ifnar1*-KO tumors (Supplemental Figure 4, B, E, and F). In B16F10 and LLC tumors, there were many fewer cells expressing PD-1 than in MC38 tumors, but still no significant differences in the percentage of CD8^+^ T cells expressing PD-1 or TIM-3 between WT and *Ifnar1*-KO tumors were found. The paucity of T cells in the KPC model precluded their analysis. Overall, our experiments uncovered no evidence that *Ifnar1* KO in cancer cells led to consistently increased numbers of CD8^+^ T cells, enhanced expression of markers for cytotoxic capacity, or reduced exhaustion in tumor-infiltrating CD8^+^ T cells.

### Ifnar1-KO cancer cells are more susceptible to CD8^+^ T cell–mediated killing.

**Figure 4:**
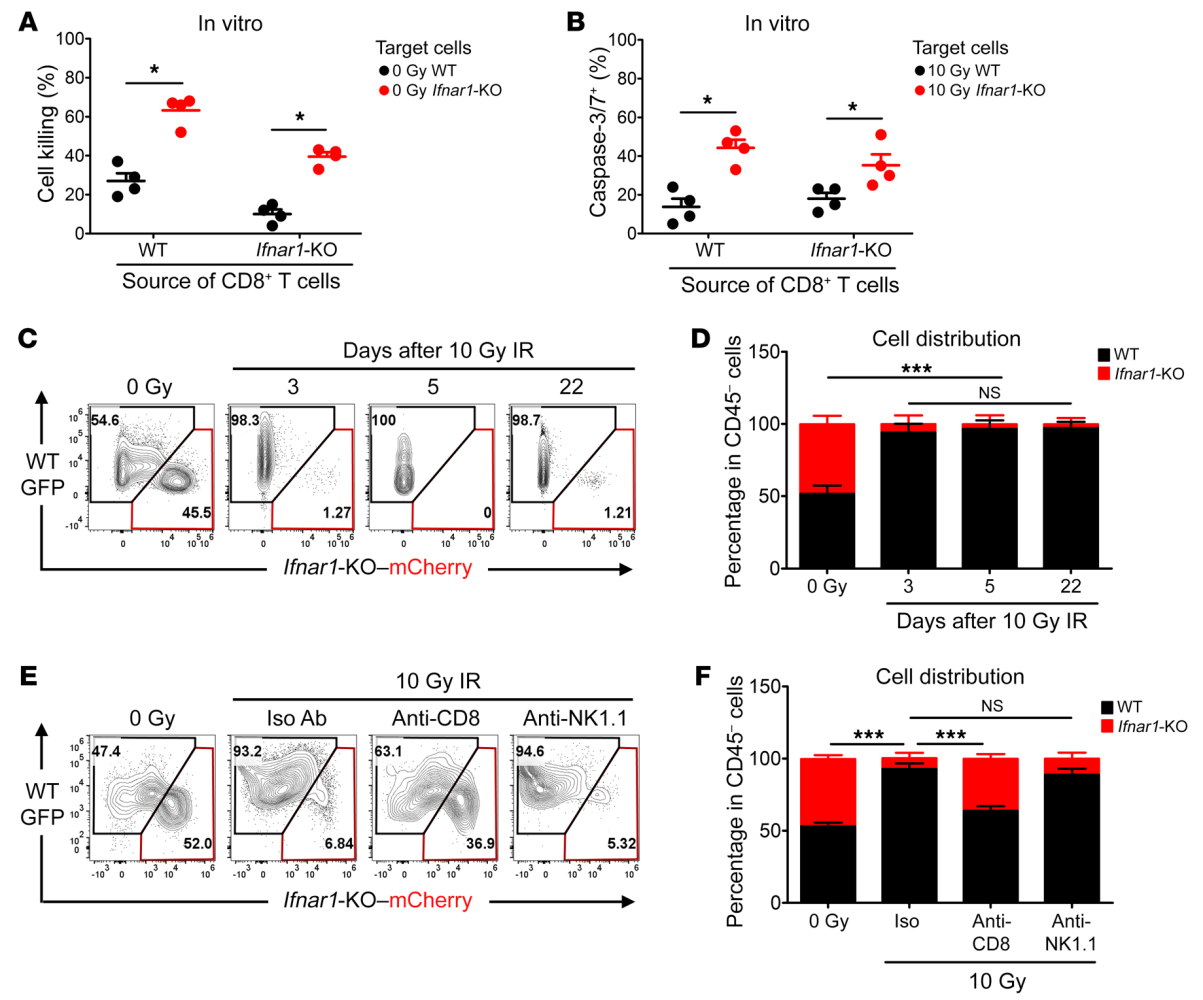
*Ifnar1-*KO MC38 tumor cells are more susceptible to CD8^+^ T cell–mediated killing. WT and *Ifnar1*-KO MC38 cells were cocultured with CD8^+^ T cells derived from either WT or *Ifnar1*-KO MC38 tumors at a ratio of CD8^+^ T cells/tumor cells of 3:2 for 48 hours. (**A**) Percentage of cell killing. *n* = 4. Irradiated MC38 cells (WT or *Ifnar1*-KO) were cocultured with CD8^+^ T cells derived from irradiated WT or *Ifnar1*-KO MC38 tumors at a ratio of 3:2. Cells in medium supplemented with Caspase-3/7 Green detection reagent were imaged with an epifluorescence microscope. (**B**) The percentage of tumor cells becoming caspase-3/7^+^ following interaction with CD8^+^ T cells was evaluated. *n* = 4. GFP-tagged WT MC38 cells and mCherry-tagged *Ifnar1*-KO MC38 cells at a ratio of 1:1 were injected subcutaneously into C57BL/6 mice. Established tumors were subjected to 0 Gy or 10 Gy IR on day 0. On days 3 and 5, and 22 for irradiated tumors, the percentages of GFP- and mCherry-positive cells in the CD45-negative population were assessed by flow cytometry (representative plot in **C** and summary in **D**). *n* =4. Tumors formed from a mixture of cells (MC38 WT-GFP + *Ifnar1*-mCherry, 1:1) were subjected to the following: 0 Gy, 10 Gy (day 0) + isotype control Abs; 10 Gy + anti-CD8 Ab; and 10 Gy + anti-NK1.1 Ab. The distribution of GFP versus mCherry cells in the CD45-negative live population on day 3 was assessed by flow cytometry (representative plot in **E** and quantification in **F**). *n* = 5. Data represent mean ± SD. Comparison of 2 means was performed by the Mann-Whitney *U* test. Comparison of means of more than 2 groups was performed by 1-way ANOVA with Tukey’s multiple-comparisons test (NS: *P* ≥ 0.05, *P < 0.05, ***P < 0.001).

Since we found little alteration in CD8^+^ T cell numbers or functional markers in *Ifnar1*-KO compared with WT tumors, we next asked whether *Ifnar1*-KO cancer cells were more susceptible to CD8^+^ T cells. In culture, WT and *Ifnar1*-KO MC38 cells were incubated with CD8^+^ T cells isolated from either WT or *Ifnar1*-KO tumors. The *Ifnar1*-KO MC38 cells were substantially more susceptible to killing by both CD8^+^ T cell populations than the WT cells (Figure 4A). Similar results were obtained with WT and *Ifnar1*-KO B16F10 cells using CD8^+^ T cells from their tumors (Supplemental Figure 5A). Since irradiation of these cancer cells leads to cell cycle arrest and lack of viability, but only low levels of apoptosis, and since T cell killing is driven by apoptosis, we used apoptosis as an endpoint to ask whether irradiated *Ifnar1*-KO cancer cells were also more susceptible to apoptosis. We found that CD8^+^ T cells isolated from irradiated MC38 tumors generated significantly increased apoptosis of irradiated *Ifnar1*-KO MC38 cells than of irradiated WT MC38 cells (Figure 4B). Thus, *Ifnar1*-KO cancer cells were more vulnerable to CD8^+^ T cell–mediated killing in culture.

Next, we asked whether there were similar susceptibility differences in the immune microenvironment in vivo. GFP-tagged WT MC38 cells and mCherry-tagged *Ifnar1*-KO MC38 cells were coinjected into C57BL/6 mice subcutaneously at a 1:1 ratio. As shown in Figure 4, C and D, these mixed tumors retained the same ratio of cell types after growth in syngeneic mice. In contrast, following IR, *Ifnar1*-KO–mCherry cells were almost completely absent from tumors, with WT-GFP cells making up the majority of the CD45-negative cells. To determine whether CD8^+^ T cells contributed to the disparity in *Ifnar1*-KO cell survival after IR, we depleted CD8^+^ T cells with Ab. This partially restored the proportion of *Ifnar1*-KO cells to that before radiation. Depletion of NK cells had no effect (Figure 4, E and F). Similar results were found in the B16F10 model (Supplemental Figure 5, B and C). Thus, in vivo as in vitro, *Ifnar1*-KO cancer cells showed greater susceptibility to CD8^+^ T cell–mediated killing than WT cells.

### Serpinb9 is the key mediator of enhanced susceptibility of Ifnar1-KO cancer cells.

**Figure 5:**
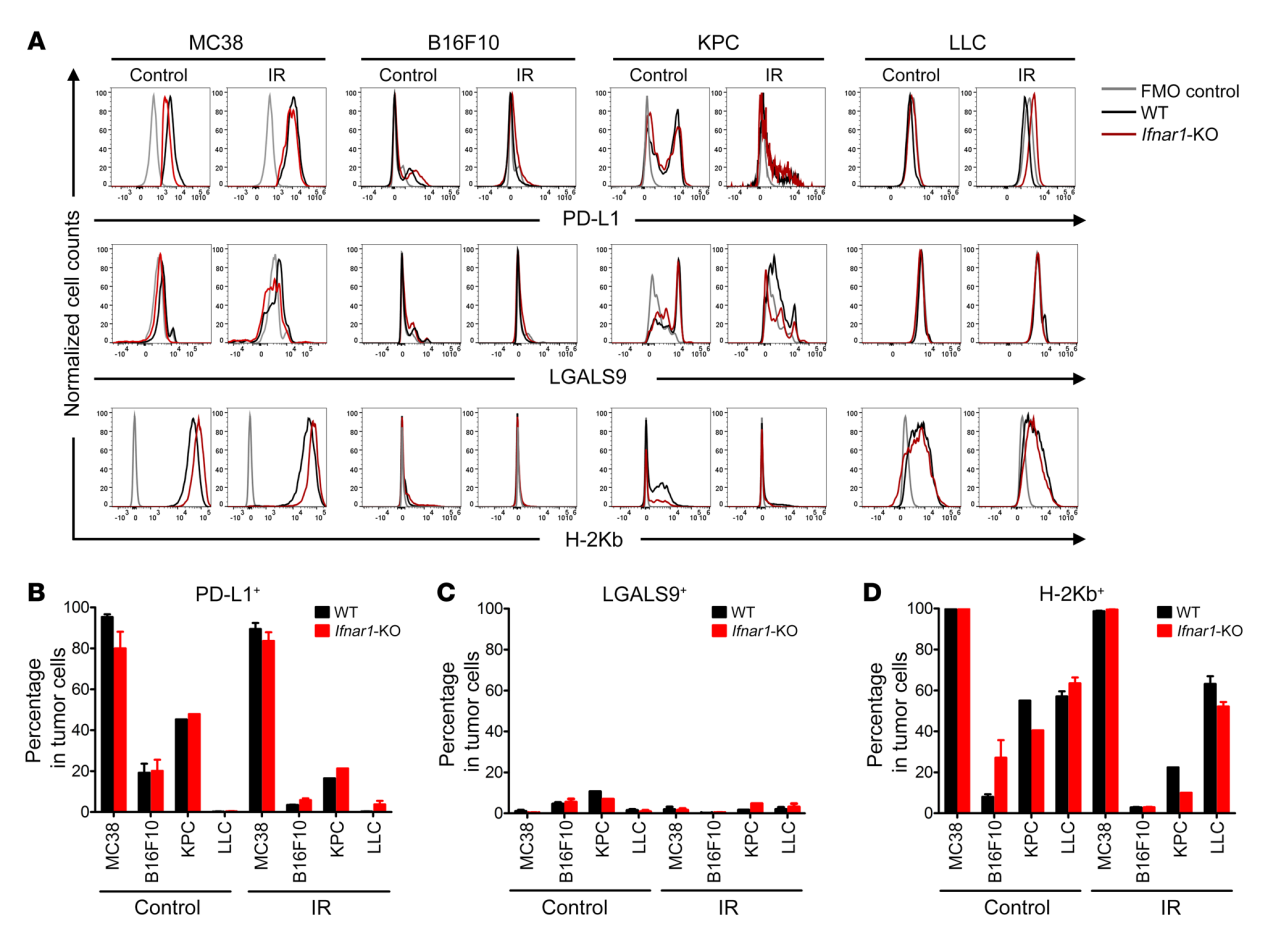
Flow cytometry profiling of candidate mediators. The abundance of PD-L1, LGALS9, and H-2Kb on the surface of cells from either WT or *Ifnar1*-KO tumors on day 4 following 0 Gy or IR administration was assessed by flow cytometry in tumors from the indicated cell lines. (**A**) Cells stained with FMO were negative controls (gray line); WT cells are represented in black and *Ifnar1*-KO in red. (**B**–**D**) Percentages of PD-L1– (CD274), LGALS9-, and H-2Kb–positive cells. *n* = 5–6, except for KPC cells, where *n* = 1 due to the necessity of pooling samples to obtain sufficient material for analysis. Data represent mean ± SD.

Cancer cell resistance to CD8^+^ T cell killing can be mediated through a variety of intrinsic mechanisms, including upregulation of immune checkpoints, granzyme inhibitors, or antiapoptotic factors, and downregulation of MHC class I molecules (31–35). To search for candidate genes underlying the enhanced susceptibility of *Ifnar1*-KO cancer cells, we generated a panel of 12 plausible candidates for an initial survey. We stipulated that a suitable candidate would be induced (or suppressed) in cancer cells by IR. Table 1 shows that 5 candidate genes were significantly induced by IR, with additional genes excluded due to failure to be either induced or expressed in less than 20% of MC38 cells (Supplemental Figure 6). We also stipulated that expression would be reduced in *Ifnar1-*KO cells compared with WT for those genes mediating resistance to killing or increased for MHC class I (here, *H2-K1*). Of the candidates examined, only *Serpinb9* met these criteria in both the MC38 and B16F10 models, and thus we concentrated our efforts on *Serpinb9* (Table 1 and Figure 5).

**Figure 6:**
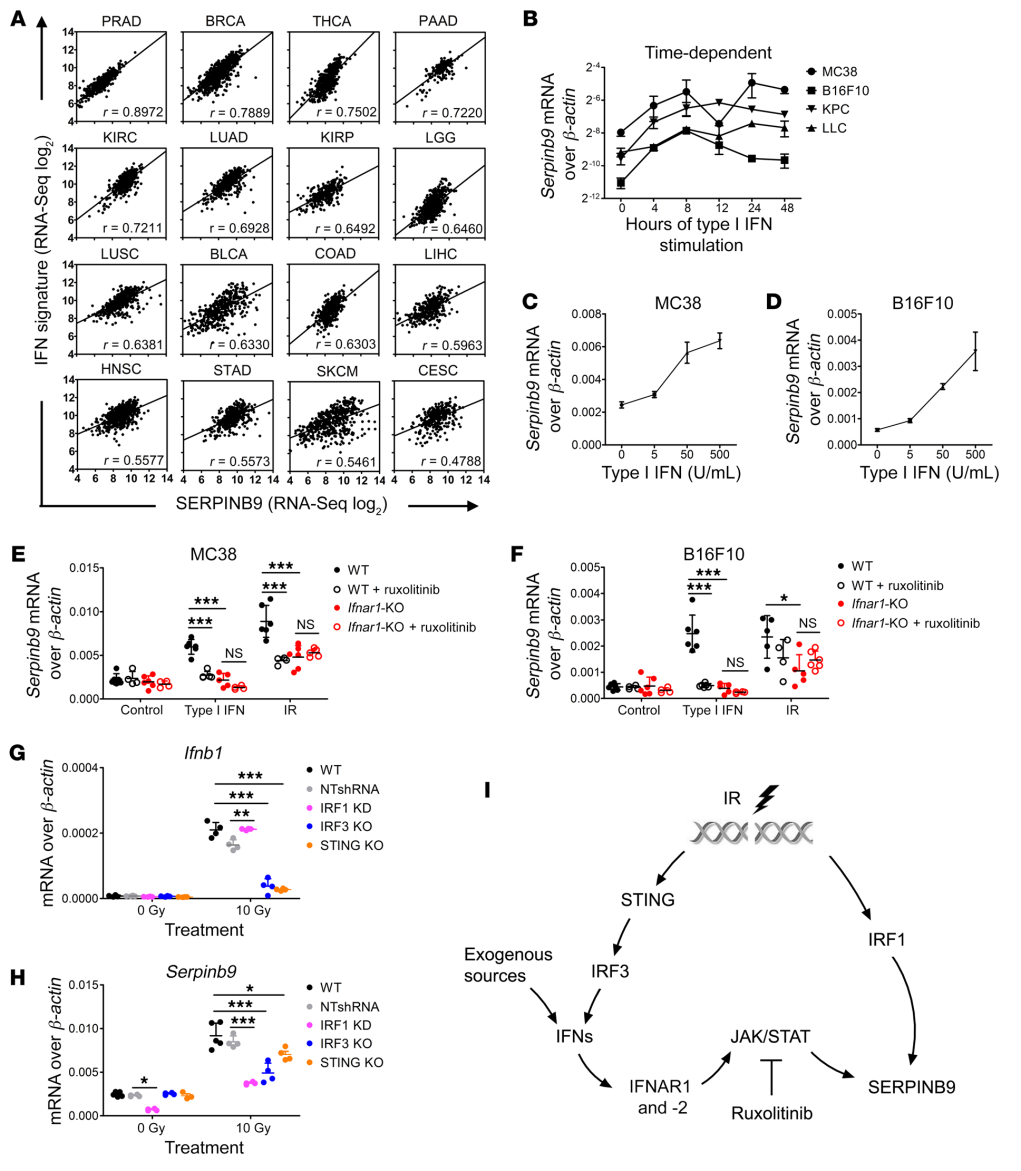
*Serpinb9* is induced by type I IFN signaling in cancer cells. (**A**) The correlation of SERPINB9 expression with IFN signature genes in 16 different human cancer types from the TCGA database was evaluated by Pearson’s correlation. Pearson’s correlation coefficients (*r*) are indicated. *P* values for all comparisons were less than 0.0001. (**B**) *Serpinb9* expression in cancer cells (MC38, B16F10, KPC, and LLC) after exposure to type I IFN (50 U/mL) over 48 hours. *n* = 3. (**C** and **D**) *Serpinb9* expression in MC38 and B16F10 cells after exposure to the indicated concentration of type I IFN (0–500 U/mL) after 4 hours. *n* = 3. (**E** and **F**) *Serpinb9* expression in MC38 and B16F10 cells (WT vs. *Ifnar1*-KO) at 4 hours after exposure to type I IFN (50 U/mL) or 72 hours after IR (10 Gy for MC38 and 20 Gy for B16F10 cells) with or without ruxolitinib. Dimethyl sulfoxide (final v/v 0.5%) or ruxolitinib (final concentration 2.5 μM) was added to the medium 1 hour before IFN or IR treatment and remained in the medium through the experiment. *n* = 4–6. (**G** and **H**) *Serpinb9* and *Ifnb1* expression in WT cells and WT MC38 cells transfected with nontargeting shRNA lentivirus (NTshRNA), and IRF1-knockdown (KD), IRF3-KO, and STING-KO MC38 cells at 72 hours after 10 Gy. *n* = 4. Gene expression was assessed by RT-qPCR. All mRNA expression levels were normalized to *β-actin*. (**I**) Scheme for induction of *Serpinb9* in cancer cells after type I IFN or IR treatment. Data represent mean ± SD. Comparison of means was performed by 1-way ANOVA with Tukey’s multiple-comparisons test (NS: *P* ≥ 0.05, *P < 0.05, **P < 0.01, ***P < 0.001).

Previous studies have shown that SERPINB9 directly inhibits granzyme B, a major effector of CD^+^ 8 T cell cytotoxicity, and leads to resistance to cytolytic T cell killing in cancer cells, making it a plausible candidate (32, 36). *SERPINB9* mRNA levels correlated with IFN gene expression signatures in a variety of cancers in the TCGA database (Figure 6A). We confirmed that *Serpinb9* RNA was induced in a dose-dependent fashion after exposure to type I IFN in the 4 cancer cell lines studied here (Figure 6, B–F). This upregulation was transient following time-limited exposure to type I IFN (Supplemental Figure 7, A and B) but was prolonged after IR of cancer cells in tissue culture over 72–120 hours (Supplemental Figure 7, C–E). Irradiated tumors had greater expression of SERPINB9 as assessed by immunohistochemistry (Supplemental Figures 8 and 9). Exposure to type I IFN failed to induce *Serpinb9* in *Ifnar1*-KO MC38 or B16F10 cells. Induction of *Serpinb9* by IR was reduced by approximately 50% in the *Ifnar1*-KO cells. Inhibition of JAK1 also abrogated the induction of *Serpinb9* by type I IFN in WT cells. Inhibition of JAK1 also reduced the induction of *Serpinb9* by IR in WT, but not in *Ifnar1*-KO, cells (Figure 6, E and F). Together these results suggest that *Serpinb9* is a downstream mediator of type I IFN signaling in cancer cells.

IR can activate STING resulting in activation of IFN transcriptional factors (IRFs) (37, 38), potentially leading to *Serpinb9* transcription. Both IRF1 and IRF3 were induced by IR in MC38 cells, and both IRF1 knockdown and IRF3 KO diminished *Serpinb9* induction after IR, but only IRF3 KO affected *Ifnb1* induction (Supplemental Figure 10 and Figure 6, G and H). *Sting* KO reduced *Serpinb9* induction after radiation. Thus, augmented levels of *Serpinb9* after IR may result from induction of type I IFN as well as IFN signaling–independent mechanisms, as schematized in Figure 6I.

To determine whether induction of *Serpinb9* contributes to resistance to antitumor immunity after IR, we used 2 approaches: genetic elimination of *Serpinb9* via CRISPR/Cas9 (SB9 KO) and *Serpinb9* overexpression in *Ifnar1*-KO and *Serpinb9-*KO cells through lentiviral transfection, which led to *Serpinb9* levels comparable to those after type I IFN induction in WT cells (Supplemental Figure 11, A and C). These cells had equivalent clonogenic cell survival after IR in culture (Supplemental Figure 11, B and D).

**Figure 7:**
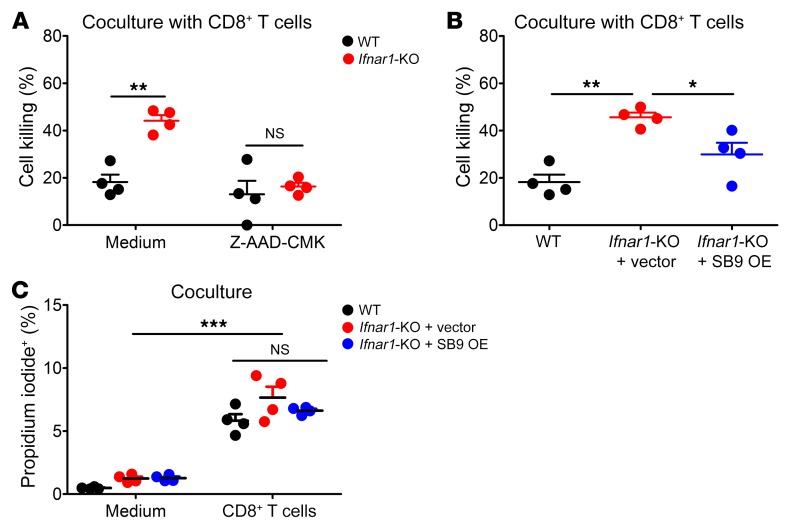
Overexpression of *Serpinb9* in *Ifnar1*-KO cancer cells reduces enhanced killing by CD8^+^ T cells in vitro. WT and *Ifnar1*-KO MC38 cells were cocultured with CD8^+^ T cells isolated from WT MC38 tumors for 48 hours with or without Z-AAD-CMK. CD8^+^ T cells were treated with Z-AAD-CMK (100 μM) for 30 minutes prior to coculture. Z-AAD-CMK remained in the medium through the experiment. (**A**) Percentage of cell killing. *n* = 4. MC38 cells (WT, *Ifnar1*-KO + vector, and *Ifnar1*-KO + *Serpinb9* overexpression [SB9 OE]) were cocultured with CD8^+^ T cells isolated from WT MC38 tumors. (**B**) Percentage of cell killing. *n* = 4. MC38 cells (WT, *Ifnar1*-KO + vector, and *Ifnar1*-KO + SB9 OE) were cocultured with either control medium or CD8^+^ T cells isolated from WT MC38 tumors for 4 hours with propidium iodide (50 μg/mL) in the medium. (**C**) Percentage of propidium iodide–positive cells following coculture. *n* = 4. Data represent mean ± SD. Comparison of means was performed by 1-way ANOVA with Tukey’s multiple-comparisons test (NS: *P* ≥ 0.05, *P < 0.05, **P < 0.01, ***P < 0.001).

CD8^+^ T cell killing is effected by granzymes including granzyme B, which gain access to the target cell through cell permeabilization by perforins. In vitro, the enhanced killing of *Ifnar1*-KO MC38 cells by CD8^+^ T cells was abolished by Z-AAD-CMK, a granzyme B–specific inhibitor (Figure 7A). *Ifnar1*-KO cells with *Serpinb9* overexpression showed a significant reduction in killing compared with cells with control vector (Figure 7B). Moreover, these cells had equivalently increased percentages of permeable cells upon coculture with CD8^+^ T cells, suggesting that abrogation of type I IFN signaling with or without *Serpinb9* overexpression in cancer cells does not affect their recognition or permeabilization by CD8^+^ T cells (Figure 7C). Thus, overexpression of *Serpinb9* in *Ifnar1*-KO cancer cells reversed their augmented susceptibility to CD8^+^ T cell–mediated killing.

**Figure 8:**
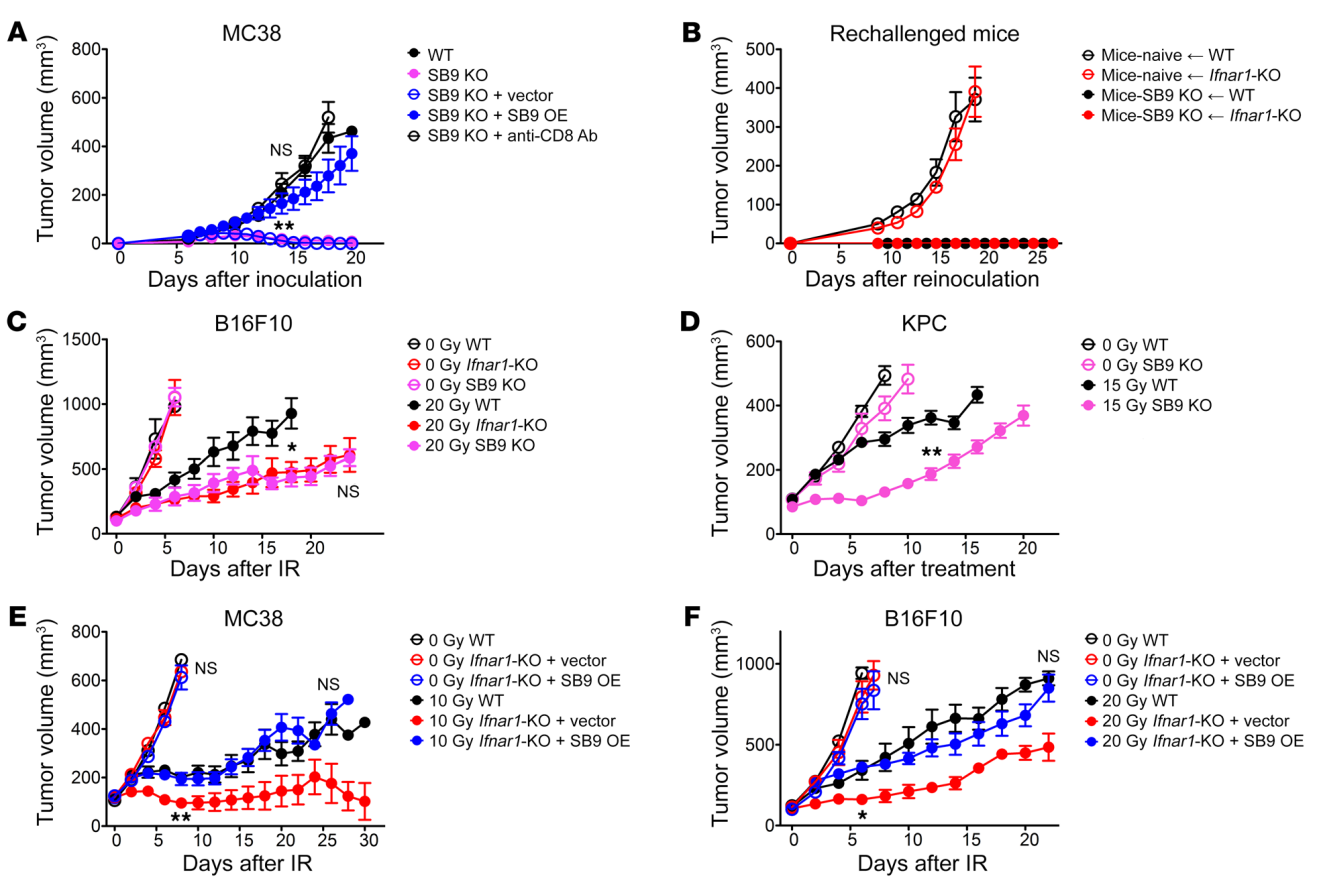
*Serpinb9* is a mediator for the enhanced response of *Ifnar1*-KO tumors to IR. (**A**) Growth of WT, SB9 KO (with or without CD8^+^ T cell depletion), SB9 KO + vector, and SB9 KO + SB9 OE MC38 tumors. *n* = 6–8. C57BL/6 mice with complete regression of SB9 KO MC38 tumors were rechallenged with WT or *Ifnar1*-KO MC38 cells on the opposite flank. (**B**) Growth of tumors following reinoculation along with growth data for WT and *Ifnar1*-KO MC38 tumors in naive mice from Figure 2G. *n* = 4–6. (**C**) Volumes of B16F10 tumors (WT, *Ifnar1*-KO, and SB9 KO) following 0 Gy or 20 Gy IR. *n* = 6–7. 20 Gy WT vs. 20 Gy SB9 KO: *P* <0.05. 20 Gy-*Ifnar1*-KO vs. 20 Gy-SB9 KO: NS. (**D**) Volumes of KPC tumors (WT and SB9 KO) following 0 Gy (*n* = 5–7) or 15 Gy (*n* = 5–6) IR. MC38 and B16F10 tumors (WT, *Ifnar1*-KO + vector, and *Ifnar1*-KO + SB9 OE) grown in C57BL6 mice were treated with 0 Gy or 10 Gy IR for MC38 and 20 Gy IR for B16F10. (**E** and **F**) Tumor growth. *n* = 5–6. Data represent mean ± SD in **A** and **B**, and mean ± SEM in **C**–**F**. Comparison of 2 means was performed by the Mann-Whitney *U* test (NS: *P* ≥ 0.05, *P < 0.05, **P < 0.01).

In vivo, *Serpinb9-*KO MC38 tumors spontaneously regressed in C57BL/6 mice after subcutaneous inoculation. Their growth was rescued by CD8^+^ T cell depletion or by reexpression of *Serpinb9* (Figure 8A). The mice that had rejected the *Serpinb9* MC38 tumors also rejected WT and *Ifnar1*-KO MC38 cells after rechallenge, suggesting that novel antigens in the KO cells were not responsible for rejection (Figure 8B).

In contrast, growth of *Serpinb9-*KO B16F10 tumors was equivalent to that of WT tumors (Figure 8C). Since *Serpinb9* was not essential for tumor growth in B16F10 cells, we used these tumors to examine the effect of IR. Following IR, *Serpinb9-*KO B16F10 tumors had a prolonged tumor growth delay equivalent to that of irradiated *Ifnar1*-KO B16F10 tumors (Figure 8C). *Serpinb9*-KO KPC tumors also exhibited a similarly enhanced response to IR (Figure 8D). Most importantly, reintroduction of *Serpinb9* expression in *Ifnar1*-KO cancer cells abrogated their enhanced response to IR. *Serpinb9* restoration did not affect *Ifnar1*-KO MC38 or B16F10 tumor growth without treatment (Figure 8, E and F). Collectively, our results reveal what we believe to be a previously unrecognized link between activation of type I IFN signaling in cancer cells and induction of *Serpinb9*, which protects cancer cells from CD8^+^ T cell–mediated cytotoxicity after irradiation.

### Ifnar1-KO tumors exhibited greater response to anti–PD-L1 than WT.

**Figure 9:**
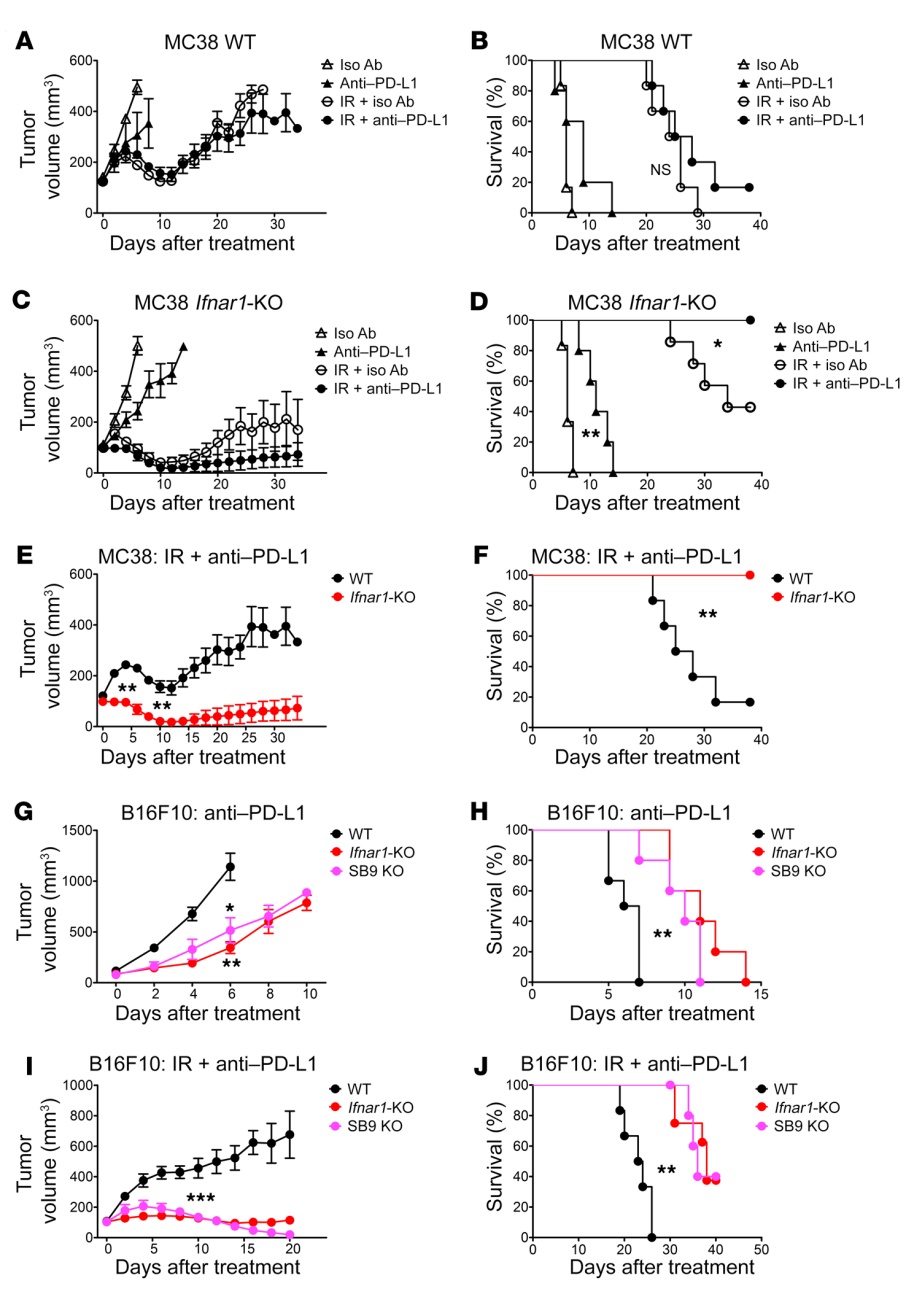
*Ifnar1*-KO or *Serpinb9*-KO tumors exhibited greater levels of response to anti–PD-L1 with or without IR than WT tumors. C57BL/6 mice bearing subcutaneous WT or *Ifnar1*-KO MC38 tumors were subjected to the following treatments: isotype control Ab; anti–PD-L1 Ab; 10 Gy IR on day 0 plus isotype control Ab; 10 Gy IR plus anti–PD-L1 Ab. Ab was administrated on days –1, 3, 7, and 11. (**A**, **C**, and **E**) Tumor volumes. (**B**, **D**, and **F**) Kaplan-Meier survival curves for mice. *n* = 5–7. C57BL/6 mice bearing subcutaneous WT, *Ifnar1*-KO, or SB9 KO B16F10 tumors received anti–PD-L1 Ab on days –1, 3, 7, and 11 with or without 20 Gy IR on day 0. (**G**–**J**) Tumor growth and survival. *n* = 5–8. Data represent mean ± SEM. Comparison of 2 means was performed with the Mann-Whitney *U* test. Survival comparisons between groups were performed using the log-rank test (NS: *P* ≥ 0.05, *P < 0.05, **P < 0.01, ***P < 0.001).

PD-L1 is known to be induced by IFNs, although this has been attributed to IFNG (39–42). Our panel of *Ifnar1*-KO cancer cells expressed PD-L1 at approximately the same extent as their WT counterparts, albeit with considerable variation among cell lines (Figure 5, A and B). Because anti–PD-L1 Abs enhance IR-induced antitumor immune responses (27, 28), we asked whether addition of anti–PD-L1 to IR would further improve the outcomes of mice bearing *Ifnar1*-KO tumors. Despite high percentages of cancer cells in the MC38 model expressing PD-L1, anti–PD-L1 only had a minor, not statistically significant, additional effect in WT MC38 tumors with or without IR (Figure 9, A and B). In contrast, anti–PD-L1 substantially extended the survival of mice bearing *Ifnar1*-KO tumors and further improved their outcomes when combined with IR (Figure 9, C and D). The majority of mice bearing *Ifnar1*-KO MC38 tumors experienced complete regression following IR plus anti–PD-L1 treatment, with significant improvement of survival compared with mice with WT tumors. Anti–PD-L1 similarly delayed tumor growth in the B16F10 model, but not in the KPC model (Figure 9, E–J, and Supplemental Figure 12). Thus, in some but not all experimental models, we observed significantly greater regression of *Ifnar1*-KO or *Serpinb9*-KO tumors with or without IR in response to anti–PD-L1.

## Discussion

The beneficial role of type I IFN signaling in immune cells for the antitumor immune response after radiation is well recognized. Less is known about the effect of type I IFN signaling in cancer cells in the context of radiotherapy. In this study, we found that intact type I IFN signaling in cancer cells was detrimental to tumor control after IR with or without anti–PD-L1 immunotherapy. Abrogation of *Ifnar1* in murine cancer cells resulted in an enhanced antitumor response after IR dependent on CD8^+^ T cells, which generally did not lead to increased numbers, augmented cytotoxic markers, or reduced exhaustion of tumor-infiltrating CD8^+^ T cells. Instead, the cancer cells themselves became more susceptible to CD8^+^ T cell–mediated killing. We identified *Serpinb9* as a critical component depleted in the *Ifnar1*-KO cancer cells. Taken together, our results reveal what we believe to be a previously undiscovered connection between activation of type I IFN signaling in cancer cells and protection from CD8^+^ T cell–mediated cytotoxicity in irradiated tumors.

Emerging data indicate that IFN signaling has paradoxical effects on tumor control (6, 43, 44). IFN signaling can enhance the generation of an antitumor response, with DCs and T cells as well recognized targets. Suppressive effects are also recognized, including induction of the immune checkpoint mediator PD-L1 (39–42, 45, 46). Radiation, through the induction of type I IFN, may amplify these effects. Benci et al. showed that prolonged IFN signaling renders cancer cells more resistant to immune checkpoint blockade with or without radiotherapy (6). Our findings suggest that intact type I IFN signaling in cancer cells reduces the benefits from the antitumor immune response after radiation, adding another layer of complexity. These results together suggest that activation of type I/II IFN signaling can play a pivotal role in immune resistance of tumor cells, but with considerable variation depending on the particular conditions.

*Serpinb9* was critical in enhancing the vulnerability of *Ifnar1*-KO cancer cells in our study, consistent with its known role as an inhibitor of granzyme B, an important mediator of T and NK cytoxicity, including killing of cancer cells (32, 36, 47–49). Pan et al performed a genome-wide CRISPR screen to identify genes whose expression can contribute to cancer cell resistance to cytotoxic T cells (50). This experiment uncovered more than 100 genes, including members of the PBAF transcriptional complex, yet with *Serpinb9* as one of the genes identified with the greatest significance. Even within a more comprehensive analysis of a correlative signature for resistance to immune checkpoint blockade in melanoma, *Serpinb9* as a single element correlated with resistance (51). *Serpinb9* expression can be triggered by a variety of mediators, including estrogen, HIF-α2, dsRNA sensing, IFN-γ, and various modulators of inflammation (52–56). Induction of *Serpinb9* by type I IFN in normal cells has been reported in the interferome (http://www.interferome.org). Even though SERPINB9 was only transiently induced by type I IFN in cancer cells, its expression was more prolonged after IR. More prolonged expression of IFN and hence SERPINB9 might result from a persistence of DNA damage after IR. Whether SERPINB9 might play a role in tumor recurrence is not known. *Serpinb9* levels at baseline as well as their upregulation in response to type I IFN or IR may serve as a common mechanism for cancer cells to survive immune attack.

Blockade of this pathway or targeting *Serpinb9* in cancer cells might be used in the context of radiation therapy to maximize benefits from the antitumor immune response. Such an approach would need to be considered carefully, since *Serpinb9* plays a role in the protection of cytotoxic effector cells themselves from granzyme B in the cytotoxic milieu and has been shown to be critical for DC-mediated antigen cross-presentation (57–61). Further investigations to discriminate between cancer cells and the host in regulation of type I IFN signaling and expression of *Serpinb9* may help in developing better stratification and understanding of how *Serpinb9* inhibition might be deployed to yield more effective and specific cancer immunotherapies.

Expression of PD-L1 in tumors was not augmented after IR in our study. Yang et al. found that intratumoral type I IFN treatment induced PD-L1 in the tumors and blocking of PD-L1 led to complete eradication of these tumors (45). Benci et al. suggest that cancer cell type I and II IFN signaling elicits an immunosuppressive effect attributed mainly to PD-L1 and LGALS9 (6). In our experiments, abrogation of type I IFN signaling in cancer cells did not affect PD-L1 expression in vivo under most of our experimental conditions, perhaps because type II IFN signaling pathways remained intact. Tumor control benefits were consistently observed, however, in *Ifnar1*-KO tumors with the addition of anti–PD-L1 treatment. There was little LGALS9 on the surface of the cell lines we examined, making it less plausible as an important mediator in our experiments. Nonetheless, a contribution of LGALS9 is likely in other settings.

A therapeutic benefit of elimination of type I IFN signaling in cancer cells was evident in vivo following irradiation and immune checkpoint blockade in our experiments. Without IR, the immunosuppressive tumor microenvironment prevailed. How IR reverses the immunosuppressive microenvironment in *Ifnar1*-KO tumors remains to be determined. Our findings shed some light on considerations for personalized cancer treatment and combining radiotherapy and immune checkpoint blockade. Radiotherapy with or without anti–PD-L1 may result in a better response in tumors with reduced type I IFN signaling in the cancer cell component and may depend on *Serpinb9* expression.

## Methods

### Mice.

Female C57BL/6 and CD-1 nude mice were purchased from Charles River Laboratory. All mice were used at age 6–8 weeks and held under specific pathogen–free conditions with humidity and temperature control. Mice were randomly divided into groups in each experiment.

### Cells.

MC38 cells were a gift from Lee Gorden (Vanderbilt University, Nashville, Tennessee, USA). KPC cells were a gift from Owen Sansom (University of Glasgow, Glasgow, United Kingdom). B16F10 and LLC cells were obtained from ATCC. Cells were cultured in DMEM or RPMI 1640 medium supplemented with 10% (v/v) FBS, 1% penicillin-streptomycin (all from Sigma-Aldrich), and 25 mM HEPES (only for B16F10). Cells were used at a passage number less than 8. Routine tests for *Mycoplasma* were negative.

### Reagents.

Universal Type I IFN Alpha (PBL) was used at 5–500 U/mL. Lipofectamine 3000 (Thermo Fisher Scientific) was used to deliver cGAMP (InvivoGen). Z-AAD-CMK (Enzo Life Sciences) was dissolved in water at 10 mM and diluted in medium at a final concentration of 100 μM. Ruxolitinib (LC Laboratories) was dissolved in DMSO at 5 mM and then diluted in medium at a final concentration of 2.5 μM.

### Abs.

Abs used in this study are described in Supplemental Table 1.

### Subcutaneous tumor model.

A total of 2.5 × 10^5^ cells were injected subcutaneously into the right flank of mice. For tumor rechallenge, 1 × 10^5^ cancer cells were injected into the left flank. Tumor sizes were measured every other day using calipers. Tumor volumes were calculated using the formula length × width^2^/2.

### In vivo radiation treatment.

Tumors received irradiation when they reached 100 mm^3^. Mice under anesthesia were shielded, with the tumor left exposed. X-ray radiation treatment (300 kV, dose rate 2.25 Gy/min) was delivered to the tumors using a GulmayRS320 irradiation system (Gulmay). Following IR, mice were culled before their tumor size exceeded the ethically acceptable limit: 500 mm^3^, or 1000 mm^3^ in the B16F10 model. Otherwise, they were observed for at least 2 months. Mice whose tumors ulcerated during the experiment were culled and excluded from the analysis. Mice with severe radiation-induced dermatitis (e.g., skin ulceration) and with complete tumor regression were sacrificed, but still included in the analysis.

### CD8^+^ T cell or NK cell depletion in C57BL/6 mice.

For CD8^+^ T cell depletion experiments, mice received anti–mouse CD8α (clone 2.43) or rat IgG2b isotype control (clone LTF-2) Ab at a dose of 10 mg/kg body weight by i.p. injection. Anti–mouse NK1.1 (clone PK136) and mouse IgG2a isotype control (C1.18.4) Ab at a dose of 20 mg/kg were used for NK cell depletion experiments. Injections were given on days –1, 2, 5, 8, and 11 (day 0: IR treatment day). The efficacy of cell depletion was validated in C57BL/6 mice bearing MC38 tumors using flow cytometry (Supplemental Figure 2).

### Anti–PD-L1 treatment in mice.

Mice received anti–mouse PD-L1 rat IgG2b (clone 10F.9G2) or rat IgG2b isotype control (clone LTF-2) Ab by i.p. injection at 10 mg/kg. Abs were administrated on days –1, 3, 7, and 11.

### Flow cytometry profiling of tumors.

Tumor samples were cut into small pieces and incubated in HBSS with collagenase II (10 mg/mL) and DNase I (2 U/mL) (1× protein transport inhibitor cocktail [Thermo Fisher Scientific] was also included when profiling cytotoxic effector cells) for 30 minutes with regular shaking at 37°C. Cell suspensions were then strained through 50-μm nylon strainers, treated with red blood cell lysis buffer (BioLegend), blocked with anti–mouse CD16/32 Abs for 5 minutes, and incubated with mixed Abs at 4°C in the dark for 45 minutes. Abs used in this study are summarized in Supplemental Table 1. Following incubation, cells were washed and fixed using Foxp3/Transcription Factor Fixation/Permeabilization working solution (Thermo Fisher Scientific) for 40 minutes at room temperature. Cells with intracellular markers in the panel were permeabilized and incubated with Abs in the dark for 45 minutes. Subsequently, these cells as well as samples without intracellular markers in the panel were washed, resuspended in PBS, and analyzed using LSR II (BD Bioscience) or Attune (Thermo Fisher Scientific). Data were analyzed using FlowJo software. CD8^+^ T cells were gated as CD45^+^, CD3ε^+^, and CD8a^+^ single live cells for profiling. Cancer cells were gated as CD45^–^, CD31^–^, and CD140a^–^ single live cells for assessment of surface marker expression. Fluorescence minus one (FMO) controls were used to discriminate negative and positive signals.

### FACS.

Single-cell suspensions were prepared and stained with mixed Abs as described above. They were sorted into designated populations using a MoFlo MLS high-speed cell sorter (Beckman Coulter).

### Isolation of CD8^+^ T cells from tumors using MACS MicroBeads.

CD8^+^ T cells were isolated from tumors using MACS CD8α (Ly-2) MicroBeads (Miltenyi Biotec) according to the manufacturer’s protocol. Briefly, single-cell suspensions derived from tumors were prepared as described above. 10^7^ Cells were resuspended in 90 μL PBS (with 0.5% BSA and 2 mM EDTA) and incubated with 10 μL CD8α MicroBeads at 4°C for 15 minutes in the dark. Following washing, cells were resuspended and added to the MACS column in the magnetic field. After washing, cells were flushed from the column and collected for downstream assays. The percentage of CD8^+^ T cells in total live cells was greater than 90% as assessed by flow cytometry.

### In vitro stimulation of CD8^+^ T cells.

CD8^+^ T cells were resuspended in RPMI medium supplemented with 10% FBS, 1% penicillin/streptomycin, 1 mM l-glutamine, and 55 μM 2-mercaptoethanol, and added to 96-well plates. Cells were then incubated with 1× cell stimulation cocktail (Thermo Fisher Scientific) composed of PMA, ionomycin, and brefeldin A for 4 hours; harvested; stained with Abs; and assessed by flow cytometry.

### In vitro killing of cancer cells by tumor-derived CD8^+^ T cells.

Cancer cells were seeded in 96-well plates 24 hours prior to coculture. Cancer cells in the well were re-counted to take account of their proliferation during culture. CD8^+^ T cells were sorted by FACS from tumors in C57BL/6 mice, added to the plated tumor cells, and maintained in the incubator. Tumor cells cultured alone were used as controls. Cocultured medium was RPMI medium supplemented with 10% FBS, 1% penicillin/streptomycin, 1 mM l-glutamine, 55 μM 2-mercaptoethanol, and 30 U/mL IL-2. Upon coculture for 48 hours, culture wells were washed extensively with PBS, fixed with 4% paraformaldehyde in PBS for 15 minutes, and stained with Hoechst (1 μg/mL) for 5 minutes. The remaining cells in the well were then quantified using a Celigo Imaging Cytometer (Nexcelom Bioscience). The killing percentage was calculated using the following formula: (cell number in control well – cell number in cocultured well)/cell number in control well × 100.

### Imaging assessment of apoptosis in cancer cells upon coculture with tumor-derived CD8^+^ T cells.

Cancer cells were tagged with mCherry via lentivirus transfection (vector: pCDH-CMV-MCS-EF1α-Hygro, System Biosciences), followed by FACS to isolate the bright population. These cells were irradiated with 10 Gy IR, immediately seeded in μ-Slide VI 0.4 channel slides (ibidi), and returned to the incubator. Forty-eight hours later, CD8^+^ T cells isolated from WT or *Ifnar1*-KO MC38 tumors by FACS on day 4 after 10 Gy IR, labeled with CMAC dye (Thermo Fisher Scientific), were added to channel slides at an effector/tumor cell (E/T) ratio of 3:2. Cancer cells were evaluated by counting cells seeded in parallel plates to take account of their altered proliferation after IR. Culture media were supplemented with IL-2 (30 U/mL) and CellEvent Caspase-3/7 Green Detection Reagent (500 nM, Thermo Fisher Scientific). Subsequently, channel slides were imaged every 15 minutes for up to 16 hours using a Nikon ECLIPSE Ti-E Inverted Microscope. During the whole course of imaging, chamber slides were maintained at 37°C with 5% CO_2_. The imaging data were transferred to an Imaris station for analysis (Bitplane). The percentage of caspase-3/7^+^ tumor cells was calculated using the following formula: number of tumor cells becoming caspase-3/7^+^ following interaction with CD8^+^ T cells/number of tumor cells interacted with CD8^+^ T cells × 100.

### IR in tissue culture.

In vitro IR was performed as described previously (12). Briefly, cells were treated with γ-rays (0.662 MeV) delivered using a ^137^Cs laboratory irradiator (IBL 637, Cisbio) at a dose rate of 0.81 Gy/min.

### Reverse transcription quantitative PCR.

Reverse transcription quantitative PCR (RT-qPCR) was performed as described previously (12). Briefly, RNA was extracted from the cells using TRIzol (Thermo Fisher Scientific) or RNeasy Mini Kit (QIAGEN), treated with DNase using a TURBO DNA-*free* Kit (Life Technologies) or an RNase-Free DNase Set (QIAGEN), and transcribed into cDNA using the High-Capacity cDNA Reverse Transcription Kit (Thermo Fisher Scientific). PCR was performed using SYBR green (Promega) with specific primers (Supplemental Table 2) or TaqMan primers/probes (Thermo Fisher Scientific, listed in Supplemental Table 3). Reactions were run on an ABI QuantStudio 5 Real-time PCR System (Thermo Fisher Scientific). The cycling conditions were as follows: (a) 50°C 2 minutes, 1 cycle; (b) 95°C 10 minutes, 1 cycle; (c) 95°C 15 seconds, followed by 60°C 60 seconds, 40 cycles. Following the amplification, the Ct values for target genes and the reference gene, *β-actin*, were recorded. Fold induction was calculated using the ΔΔCt method.

### Generation of KO cell lines using CRISPR.

Gene KO using CRISPR/Cas9 in cell lines was performed as described previously (12). Briefly, a pair of guide RNA (gRNA) primers (Supplemental Table 4) targeting a region within exons were designed using the Feng Zhang laboratory’s CRISPR Design (https://zlab.bio/guide-design-resources). These primers were cloned into Nickase Cas9 plasmids pSpCas9n (BB)-2A-GFP (PX461) and pSpCas9n (BB)-2A-Puro (PX462) (gifts from Feng Zhang through Addgene, plasmids 48140 and 62987, respectively; according to a published protocol, ref. 62). The plasmid cloning was verified by sequencing using the U6 primer (5′-GAGGGCCTATTTCCCATGATTCC-3′). Cells were then transfected with PX461–gRNA-1 and PX462–gRNA-2 plasmids simultaneously using Lipofectamine 3000 Transfection Reagent (Thermo Fisher Scientific) according to the manufacturer’s protocol. Following transfection, cells were selected with culture medium containing puromycin (1–2 μg/mL) for 24–48 hours. Cells that remained after selection were used to generate single-cell clones via serial dilution or FACS. Single-cell clones with gene KO were selected and validated using Western blot analyses or functional assays. Pooled WT clones or cells transfected with empty plasmids when WT clones were not available were used as controls.

### Gene knockdown using shRNA.

Cells were transfected with nontarget shRNA control (SHC016V-1EA) or shRNA targeting mouse IRF1 (SHCLNV-NM_008390 [TRCN0000077441]) lentivirus particles (both from Sigma-Aldrich) using SureENTRY reagent (QIAGEN) according to the manufacturer’s protocol. Transfected tumor cells were selected with puromycin (1.5 μg/mL) for 10–14 days. The efficiency of knockdown was assessed using immunoblotting.

### Serpinb9 expression via lentiviral transfection.

Full-length mouse *Serpinb9* cDNA was amplified from the pCMV-SPORT6 vector (Dharmacon, accession BC029900, clone 4925100) using primers (forward, 5′-GCTCTAGAATGAATACTCTGTCTGAAGG-3′, reverse, 5′-CGGGATCCTGGAGATGAGAACCTGCCAC-3′) and inserted into the XbaI and BamHI sites of pCDH-CMV-MCS-EF1-Hygro vector (System Biosciences). The cloning of the vector was validated by sequencing. This vector together with pMD2.G and pCMV-dR8.74 were used for transfection of 293T cells to produce lentiviral particles. Viral particles collected from the culture supernatants were used for transfection of cancer cells using SureENTRY.

### Clonogenic assay.

Clonogenic assay was performed as previously described (12).

### Cell proliferation assay.

Cell proliferation was assessed using WST-1 (Sigma-Aldrich) according to the manufacturer’s protocol.

### Assessment of caspase-3/7 in cells via flow cytometry.

The presence of caspase-3/7 in cells was assessed using CellEvent Caspase-3/7 Green Detection Reagent according to the manufacturer’s protocol.

### Assessment of cell cycle in cells using propidium iodide.

Cell cycle distribution in cells was assessed using a Propidium Iodide Flow Cytometry kit (Abcam) according to the manufacturer’s protocol.

### ELISA.

Cell culture supernatants were collected from MC38 cells at 72 hours after mock treatment or 10 Gy IR. Mouse IFN-β protein levels in these supernatants were measured using the mouse IFN-beta ELISA Kit (R&D Systems) according to manufacturer’s protocol.

### Immunoblotting.

Immunoblotting was performed as previously described (12). Abs used in this study are listed in Supplemental Table 1.

### Fluorescence staining and confocal imaging.

Fluorescence staining was performed as previously described (12). Abs used in this study are listed in Supplemental Table 1.

### Immunohistochemical staining.

Mouse tumors were frozen, sectioned, fixed in acetone, blocked with hydrogen peroxide and normal horse serum (Vector Laboratories), and then incubated with primary Abs at 4°C overnight. Following washing, they were developed using an HRP Anti-Rabbit IgG Polymer Detection Kit and DAB Substrate Kit (both from Vector Laboratories) and counterstained with hematoxylin. Stained sections were dehydrated, cleared, mounted, and scanned in an Aperio slide scanner (Leica Biosystems). Images were processed using ImageScope (Leica) and analyzed with ImageJ (NIH).

### Analysis of gene expression correlation between SERPINB9 and IFN signatures in TCGA data.

TCGA gene expression data (RNA-Seq V2 RSEM) of 10 IFN signature genes (*CSF2RB*, *CD86*, *CD69*, *GBP1*, *BIRC3*, *RUNX3*, *STAT1*, *SERPING1*, *IFI16*, and *IRF1*; derived from Supplemental Table 5) and *SERPINB9* in 16 different human cancer types were downloaded via cBioPortal for Cancer Genomics (http://www.cbioportal.org/) and log_2_ transformed. The numbers of cases for each cancer type were as follows: prostate adenocarcinoma (PRAD), *n* = 498; breast invasive carcinoma (BRCA), *n* = 1100; thyroid carcinoma (THCA), *n* = 509; pancreatic adenocarcinoma (PAAD), *n* = 178; kidney renal clear cell carcinoma (KIRC), *n* = 446; lung adenocarcinoma (LUAD), *n* = 517; kidney renal papillary cell carcinoma (KIRP), *n* = 291; brain lower grade glioma (LGG), *n* = 530; lung squamous cell carcinoma (LUSC), *n* = 501; bladder urothelial carcinoma (BLCA), *n* = 408; colorectal adenocarcinoma (COAD), *n* = 382; liver hepatocellular carcinoma (LIHC), *n* = 373; head and neck squamous cell carcinoma (HNSC), *n* = 522; stomach adenocarcinoma (STAD), *n* = 415; skin cutaneous melanoma (SKCM), *n* = 472; and cervical squamous cell carcinoma and endocervical adenocarcionoma (CESC), *n* = 306. The correlation between IFN signature generated as the mean expression level of these 10 signature genes and *SERPINB9* was evaluated by Pearson’s correlation. Pearson’s correlation coefficients (*r*) are indicated. *P* values for all correlations were less than 0.0001.

### Statistics.

All values in this study represent mean ± SD, with the exception that bars in the growth curves for tumors show mean ± SEM. Comparisons were performed using unpaired 2-tailed Student’s *t* test when data were normally distributed (evaluated using D’Agostino-Pearson omnibus normality test) and Mann-Whitney *U* test when they were not or their normality could not be evaluated due to small sample size. Comparison of means of more than 2 groups was performed by 1-way ANOVA with Tukey’s multiple-comparisons test. Survival time was measured from the beginning of IR treatment until the date mice were culled. Mice culled due to radiation-induced dermatitis with complete tumor regression were survival censored and counted as tumor free. Survival curves for different groups of mice were generated using the Kaplan-Meier method. The log-rank test was used to compare the median survival times. *P* < 0.05 was considered significant. All graphs were plotted using GraphPad Prism 5.

### Study approval.

All animal experiments were conducted in accordance with the United Kingdom Animals (Scientific Procedures) Act 1986 as amended (Amendment Regulations 2012 [SI 2012/3039]), under the authority of a UK Home Office Project Licence (PPL 30/2922 and PCDCAFDE0), with local ethical approval from the University of Oxford Animal Welfare and Ethical Review Panel.

## Author contributions

JC conceived, designed, performed, and analyzed experiments and wrote the manuscript. YC, BM, JK, and JAFV performed experiments. RJM conceived, designed, and supervised the project and wrote the manuscript.

## Supplementary Material

Supplemental data

## Figures and Tables

**Table 1:**
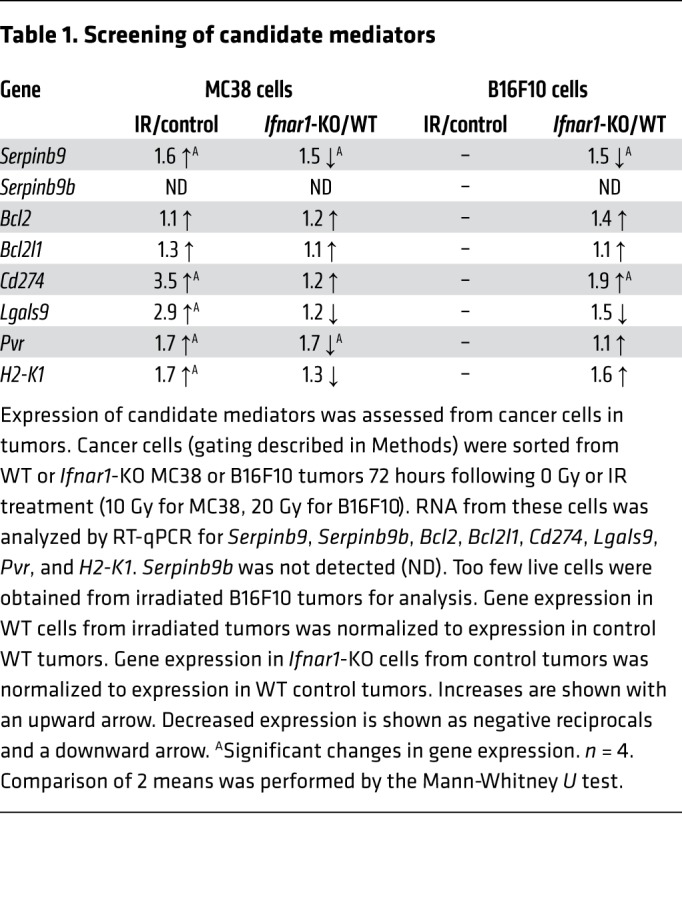
Screening of candidate mediators

## Version 1 09/04/2019

Electronic publication

## Version 2 10/01/2019

Print issue publication
