# Supplementary material for: Type I IFN protects cancer cells from CD8+ T cell–mediated cytotoxicity after radiation
Source: J Clin Invest. 2019 Sep 4;129(10):4224–38. doi: 10.1172/JCI127458 (PMC6763250; doi:10.1172/JCI127458)
Supplement: Supplemental data [file jci-129-127458-s357.pdf]

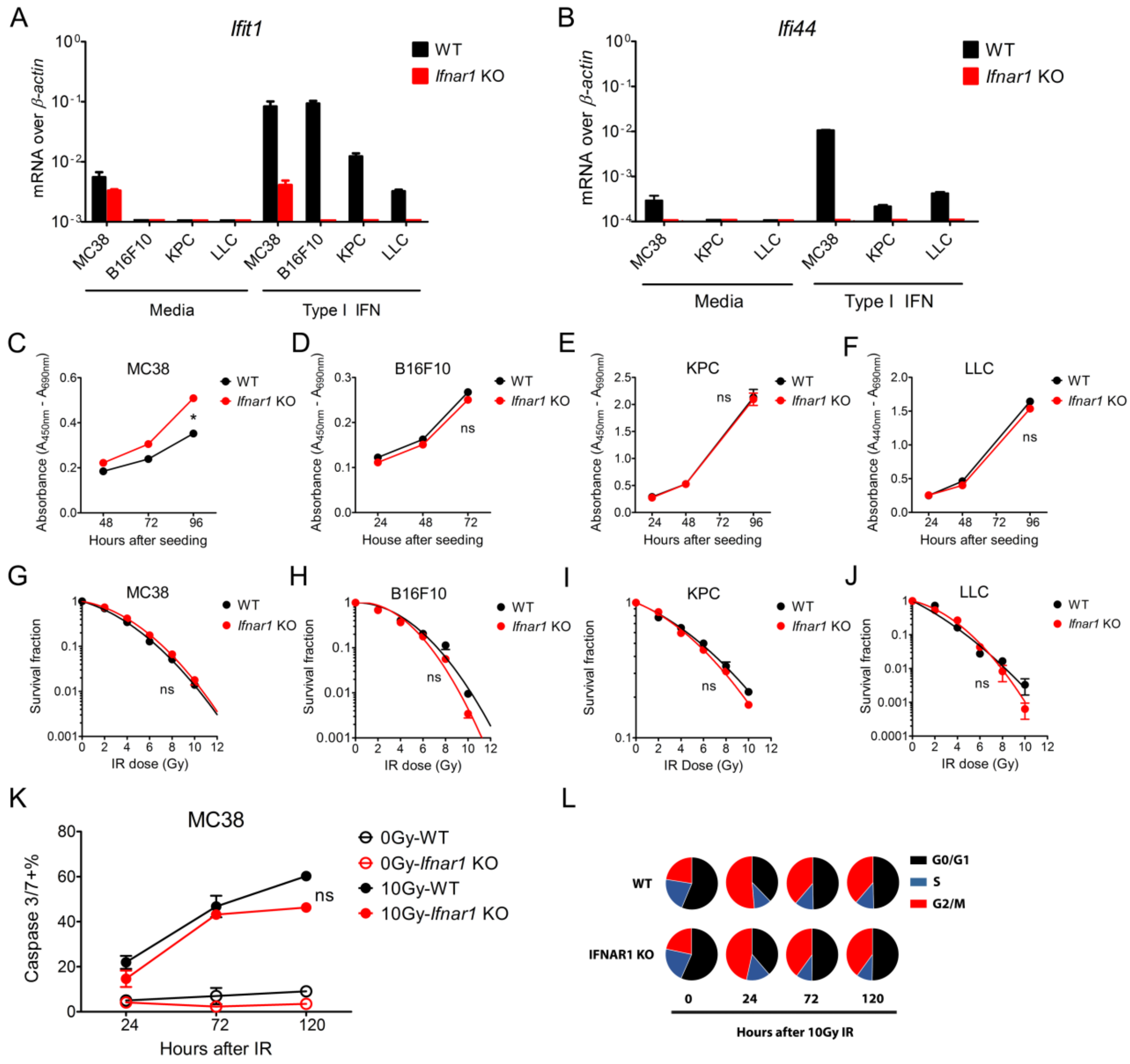

A
Ifit1
mRNA over β-actin
WT
Ifnar1 KO
MC38
B16F10
KPC
LLC
Media
Type I IFN
B
Ifi44
C
MC38
Absorbance (A450nm - A690nm)
Hours after seeding
ns
D
B16F10
House after seeding
E
KPC
F
LLC
Absorbance (A440nm - A690nm)
G
Survival fraction
IR dose (Gy)
H
I
IR Dose (Gy)
J
K
Caspase 3/7+%
0Gy-WT
0Gy-Ifnar1 KO
10Gy-WT
10Gy-Ifnar1 KO
Hours after IR
L
WT
IFNAR1 KO
G0/G1
S
G2/M
Hours after 10Gy IR

Figure S1. The radiosensitivity and proliferation in culture of WT and *Ifnar1* KO MC38, B16F10, KPC and LLC cells are similar. *Ifnar1* was genetically eliminated in the indicated cancer cell lines using CRISPR/Cas9. WT and *Ifnar1* KO MC38, B16F10, KPC and LLC cells were exposed to type I IFN (50 U/ml) for 4 hours. *Ifit1* **(A)** and *Ifi44* **(B)** expression in these cells was evaluated by RT-qPCR. N = 3. The proliferation of the indicated cells in tissue culture was evaluated using a WST-1 assay and plotted in **(C-F)**. N = 5-8. Clonogenic survival of the indicated cells was shown in **(G-J)**. N = 3. The percentages of apoptotic MC38 cells (WT and *Ifnar1* KO) at indicated time points following 10 Gy IR was determined by flow cytometry with Caspase 3/7-FITC staining **(K)**. N = 3. The distribution of cell cycle phases (G0/G1, S and G2/M phases) in MC38 cells (WT and *Ifnar1* KO) at indicated times following 10 Gy IR was determined using flow cytometry after Propidium Iodide (PI) staining **(L)**. N = 1. Data represent mean ± SD. Comparison of two means was performed by the Mann-Whitney U test (ns: $P > 0.05$, * $P < 0.05$, ** $P < 0.01$, *** $P < 0.001$).

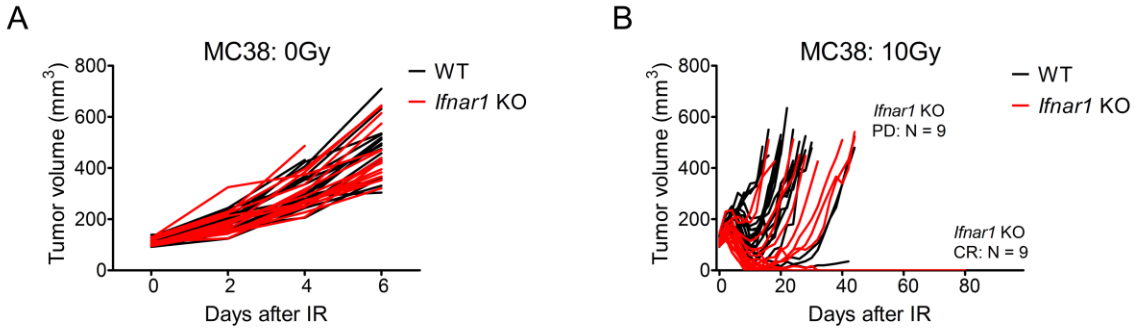

Figure S2. Growth of individual tumors. Growth of individual tumors (WT and *Ifnar1* KO MC38) following 0Gy or 10Gy IR (data from **Figure 1A**) are shown in **(A)** and **(B)**. N = 18 for the WT group and 17 for the Ifnar1 KO group in **(A)**. N = 20 for the WT group and 18 for the Ifnar1 KO group in **(B)**. The number of tumors with progressive disease (PD) or complete regression (CR) in the *Ifnar1* KO group of **(B)** is indicated.

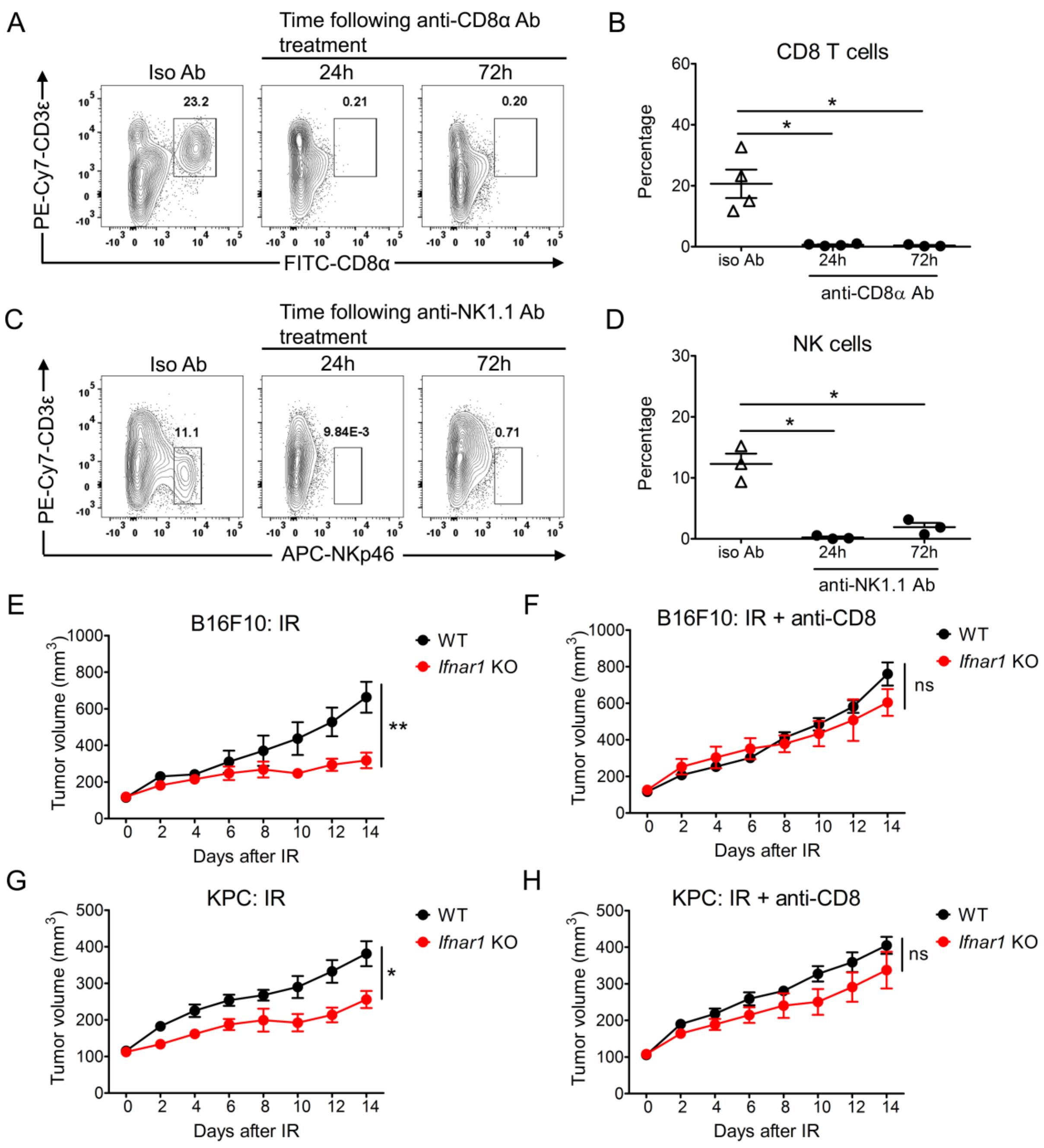
A
Time following anti-CD8α Ab treatment
Iso Ab
24h
72h
23.2
0.21
0.20
PE-Cy7-CD3ε
FITC-CD8α
B
CD8 T cells
Percentage
iso Ab
24h
72h
anti-CD8α Ab
C
Time following anti-NK1.1 Ab treatment
Iso Ab
24h
72h
11.1
9.84E-3
0.71
PE-Cy7-CD3ε
APC-NKp46
D
NK cells
Percentage
iso Ab
24h
72h
anti-NK1.1 Ab
E
B16F10: IR
Tumor volume (mm3)
Days after IR
WT
Ifnar1 KO
**
F
B16F10: IR + anti-CD8
Tumor volume (mm3)
Days after IR
WT
Ifnar1 KO
ns
G
KPC: IR
Tumor volume (mm3)
Days after IR
WT
Ifnar1 KO
*
H
KPC: IR + anti-CD8
Tumor volume (mm3)
Days after IR
WT
Ifnar1 KO
ns

Figure S3. The enhanced response of *Ifnar1* KO tumors to IR in B16F10 and KPC models is mediated by CD8 T cells. C57BL/6 mice bearing MC38 tumors received rat IgG2b isotype control antibody, anti- CD8 Ab, mouse IgG2a isotype control Ab, or anti-NK1.1 IgG2a Ab. 24 and 72 hours following injection, the percentages of tumor-infiltrating CD8 T cells (representative plot in **(A)** and quantification in **(B)**) or NK cells (representative plot in **(C)** and quantification in **(D)**) out of total live CD45 positive cells was assessed by flow cytometry. Data represent mean ± SD. Subcutaneous tumors from WT or *Ifnar1* KO B16F10, or KPC tumors in C57BL/6 mice were subjected to 20Gy and 15Gy IR respectively, on day 0. Abs were administered on days -1, 2, 5, 8 and 11. Following IR, tumor volumes were measured every other day using callipers and summarized in **(E-H)**. The growth of WT and *Ifnar1* KO tumors following IR is plotted in **(E)** and **(G)**. N = 5-6. Data represent mean ± SEM. Comparison of two means was performed by the Mann-Whitney U test (ns: $P > 0.05$, * $P < 0.05$, ** $P< 0.01$, *** $P< 0.001$).

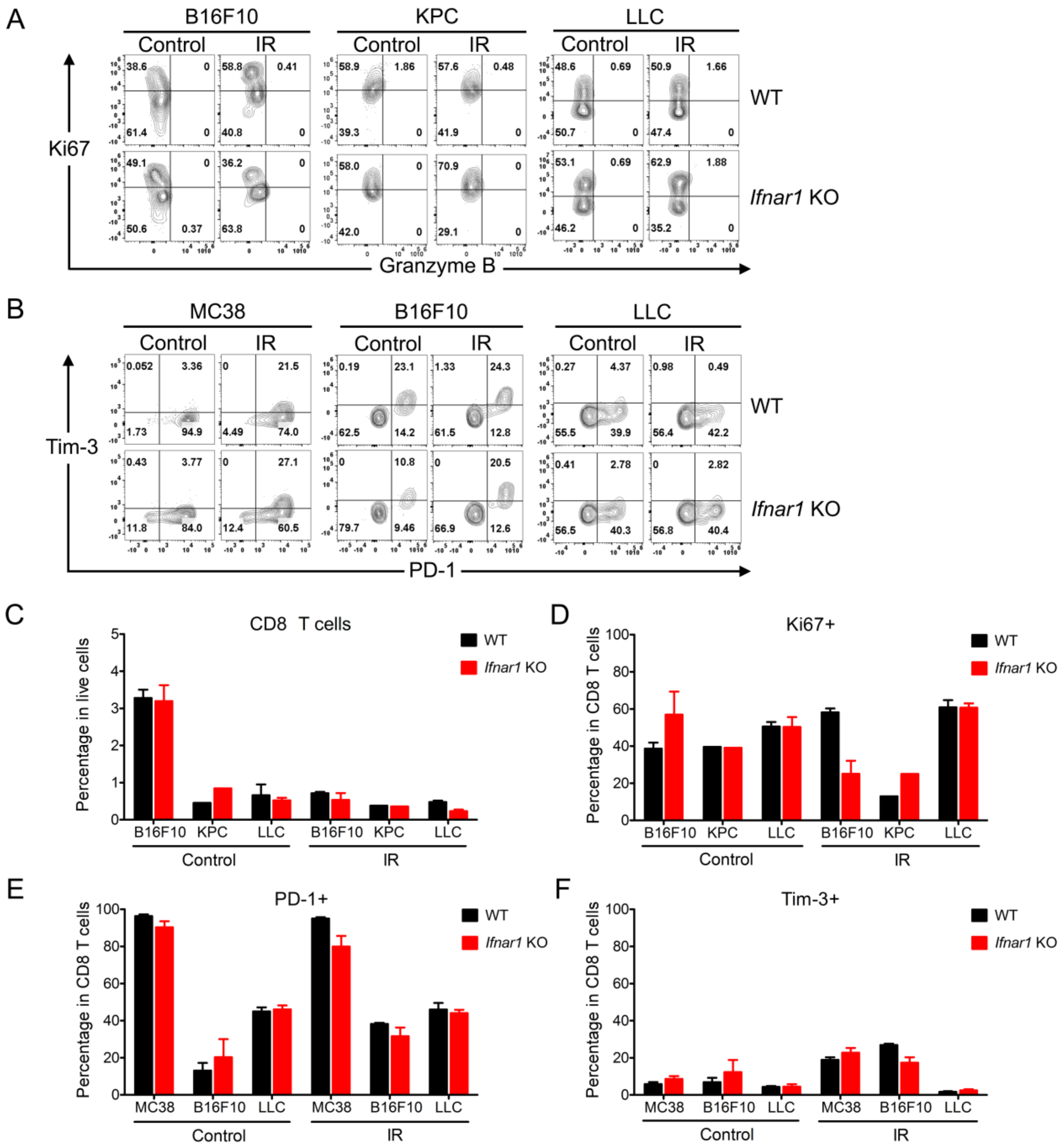
A
B16F10
KPC
LLC
Control
IR
WT
Ifnar1 KO
Ki67
Granzyme B
B
MC38
B16F10
LLC
Control
IR
Tim-3
PD-1
C
CD8 T cells
Percentage in live cells
D
Ki67+
Percentage in CD8 T cells
E
PD-1+
F
Tim-3+
WT
Ifnar1 KO

Figure S4. Infiltrating CD8 T cells from *Ifnar1* KO tumors did not exhibit either increased cytotoxic markers or reduced exhaustion. Tumors in C57BL/6 mice from MC38, B16F10, KPC or LLC cells (WT and *Ifnar1* KO) were subjected to 0 Gy, 10Gy (MC38 and LLC), 15Gy (KPC) and 20Gy (B16F10) IR. At day 4 after IR, tumors were harvested and disaggregated for CD8 T cell profiling using flow cytometry. Representative plots for the characterization of gated CD8 T cells are shown in **(A)** with Granzyme B plotted against Ki67 and in **(B)** Tim-3 plotted against Pd-l1. **(C)** shows the percentage of CD8 T cells out of total live cells. The percentages of Ki67 **(D)**, PD-1**(E)** and Tim-3 **(F)** positive CD8 T cells in WT or *Ifnar1* KO tumors with or without IR are shown. N = 3-6 with the exception of KPC which only had one sample and was pooled from 3 individual tumors to obtain sufficient materials for analysis in **(A**-**C)** and was not analysed further. Data represent mean ± SD and could not be calculated for KPC. Comparison of two means was performed by the Mann-Whitney U test (ns: $P > 0.05$, * $P < 0.05$, ** $P< 0.01$, *** $P< 0.001$).

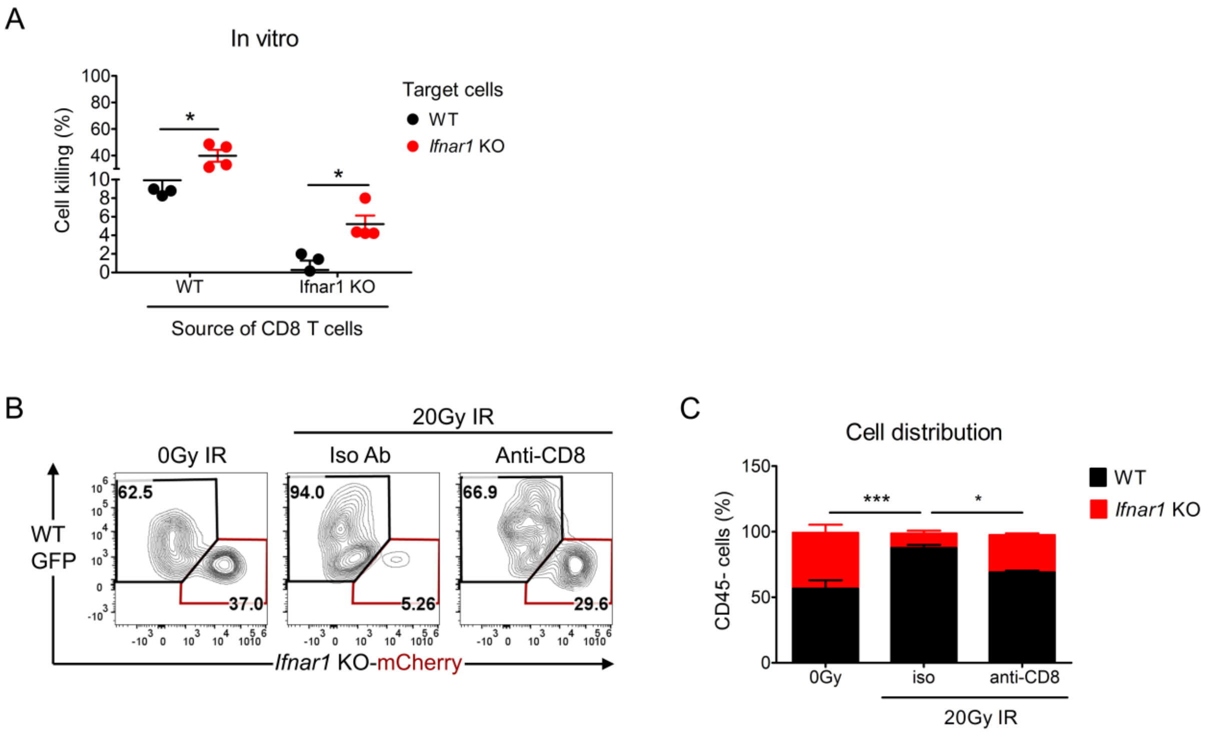
A
In vitro
Cell killing (%)
Target cells
WT
Ifnar1 KO
WT
Ifnar1 KO
Source of CD8 T cells
B
20Gy IR
0Gy IR
Iso Ab
Anti-CD8
WT GFP
62.5
37.0
94.0
5.26
66.9
29.6
Ifnar1 KO-mCherry
C
Cell distribution
CD45- cells (%)
WT
Ifnar1 KO
0Gy
iso
anti-CD8
20Gy IR

Figure S5 *Ifnar1* KO B16F10 cells are more vulnerable to CD8 T cell-mediated cytotoxicity. WT and *Ifnar1* KO B16F10 cells were co-cultured with CD8 T cells derived from either WT or *Ifnar1* KO B16F10 tumors at ratio of CD8 T cells: tumor cell ratio of 3:2 for 48 hours. The percentage of cell killing was quantified in **(A)**. N = 4. GFP-tagged WT B16F10 cells were mixed with mCherry-tagged *Ifnar1* KO B16F10 cells at a ratio of 1:1 and injected into C57BL/6 mice subcutaneously. Established tumors were subjected to the following treatments: 0Gy, 20 Gy (day 0) + isotype control Abs and 20 Gy + anti-CD8 Abs. Ab was administrated on day -1 and 3. The distribution of WT-GFP vs *Ifnar1* KO-mCherry MC38 cells in the CD45 negative live cell population at day 6 was assessed by flow cytometry (representative plot in **(B)** and quantification in **(C)**). N = 4. Data represent mean ± SD. Comparison of two means was performed by the Mann-Whitney U test. Comparison of means was performed by one-way ANOVA with Tukey's multiple-comparisons test (ns: $P > 0.05$, * $P < 0.05$, ** $P< 0.01$, *** $P< 0.001$).

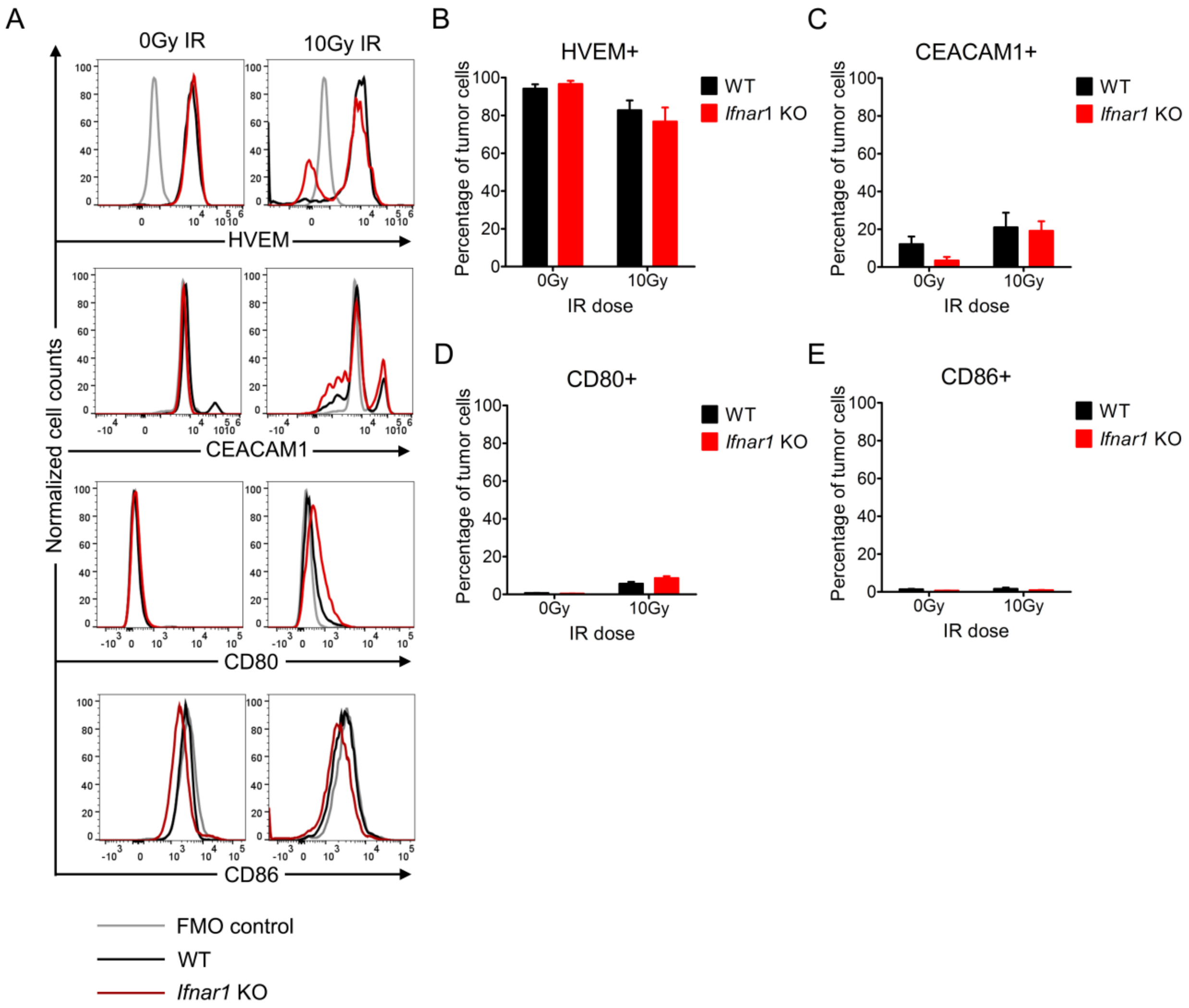
A
0Gy IR
10Gy IR
Normalized cell counts
HVEM
CEACAM1
CD80
CD86
FMO control
WT
Ifnar1 KO
B
HVEM+
C
CEACAM1+
D
CD80+
E
CD86+
Percentage of tumor cells
0Gy
10Gy
IR dose
WT
Ifnar1 KO

Figure S6 Flow cytometry profiling of immunomodulators on the surface of MC38 cells in vivo with or without IR. WT or *Ifnar1* KO MC38 tumors in C57BL/6 mice were subjected to 0 Gy or 10 Gy IR (day 0). At day 4 following IR, tumors were harvested, disaggregated, and assessed by flow cytometry. Tumor cells were gated as Alexa Fluor 700-CD45 negative, APC-CD31 negative, APC-CD140a negative live cells. HVEM, CEACAM1, CD80 and CD86 were assessed (representative plot in **(A)**). Cells stained with FMO control Ab were used as negative controls (grey line). WT MC38 cells in black and *Ifnar1* KO MC38 in red. The percentages of positive cells are summarized in **(B**-**E)**. N = 5. Data represent mean ± SD. Comparison of two means was performed by the Mann-Whitney U test (ns: $P > 0.05$, * $P < 0.05$, ** $P< 0.01$, *** $P< 0.001$).

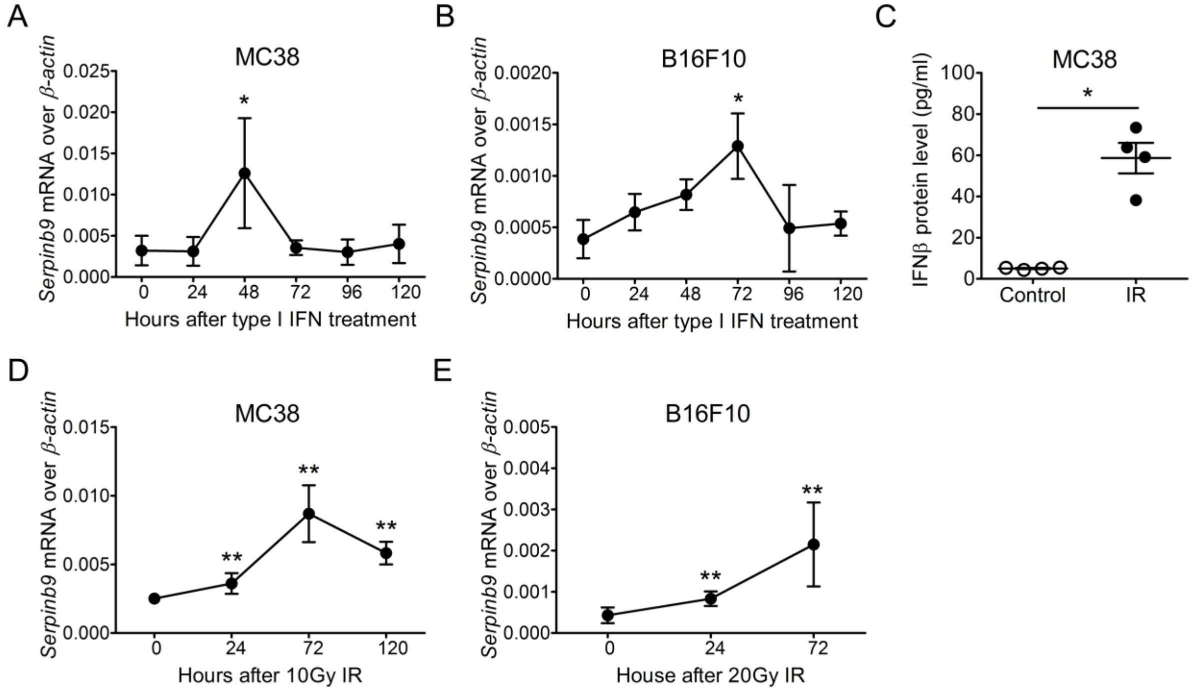

Figure S7. Induction of *Serpinb9* in MC38 and B16F10 cells after exposure to type I IFN or IR in vitro. (A) and **(B)**: *Serpinb9* expression in MC38 and B16F10 cells at a range of time (0-120 hours) after exposure to type I IFN (250 U/ml) for 4 hours. N = 4. **(C)**: IFNβ protein level in tissue culture supernatants of MC38 cells was measured by ELISA at 72 hours after 0 or 10 Gy IR. N = 4. **(D)** and **(E)**: *Serpinb9* expression in MC38 and B16F10 cells at a range of time (0-72 hours) after exposure to IR (10Gy for MC38 and 20Gy for B16F10). N = 6 in **(D)** and 5 in **(E)**. Data represent mean ± SD. Serpinb9 expression in cells following treatment was compared with that at baseline (0h). Comparison of two means was performed by the Mann-Whitney U test (ns: $P > 0.05$, * $P < 0.05$, ** $P< 0.01$, *** $P< 0.001$).

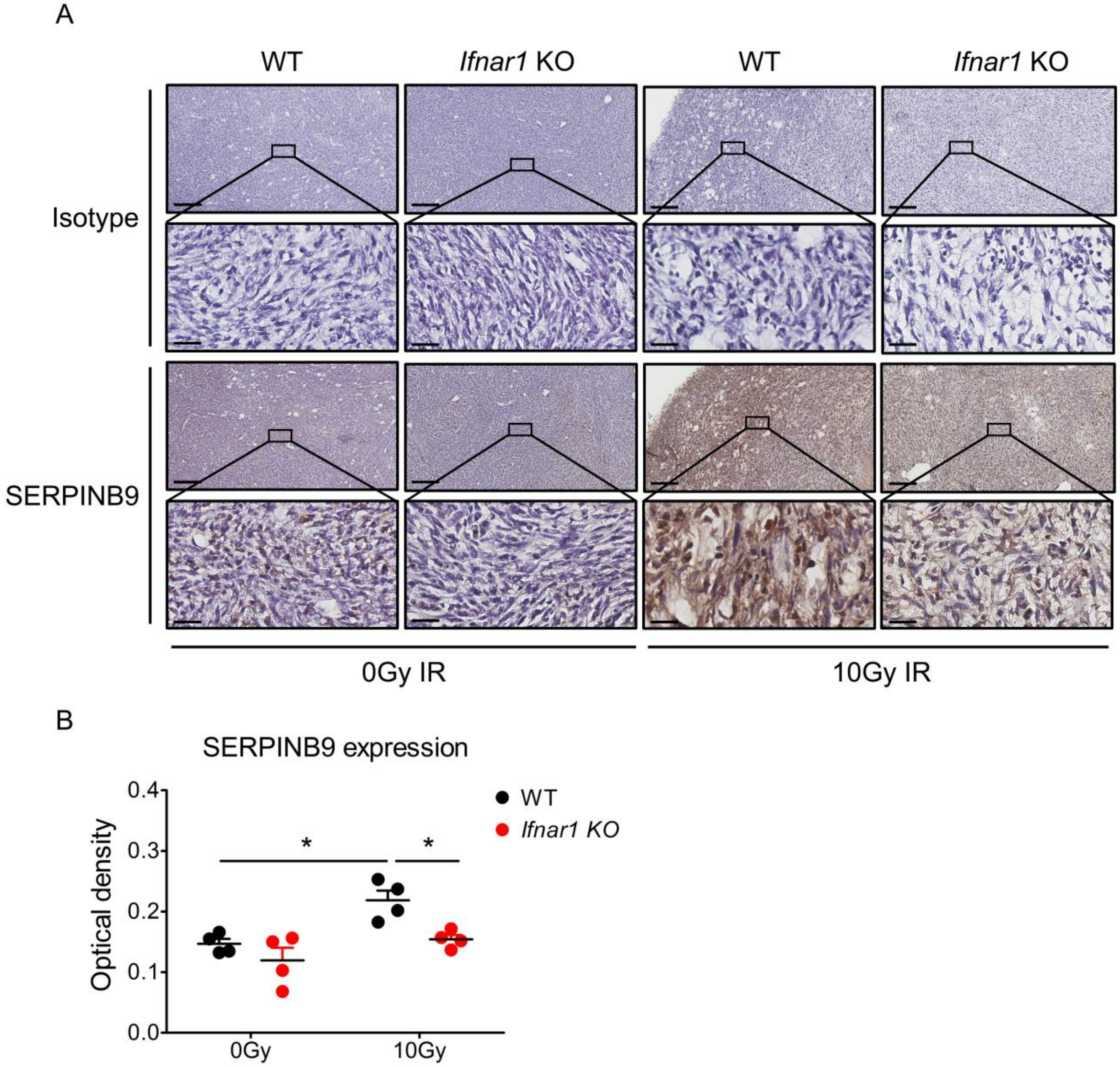
A
WT
Ifnar1 KO
WT
Ifnar1 KO
Isotype
SERPINB9
0Gy IR
10Gy IR
B
SERPINB9 expression
Optical density
0.4
0.3
0.2
0.1
0.0
0Gy
10Gy
*
*
WT
Ifnar1 KO

Figure S8. Abrogation of type I IFN signaling in cancer cells reduced the induction of SERPINB9 expression by IR in MC38 tumor sections. WT and *Ifnar1* KO MC38 tumors in C57BL/6 mice were collected at day 4 following 0 Gy or 10 Gy IR. Expression of SERPINB9 in these tumors was assessed by immunohistochemical staining. Representative images are shown in **(A)**. Tissue sections were staining with either rabbit isotype control Ab (the first and second rows) or rabbit anti-SERPINB9 Ab (the third and fourth rows). The staining was developed by Horseradish peroxidase/ 3,3'-Diaminobenzidine (brown) with hematoxylin counterstain (blue-purple). The scale bar is 200 μm for the first and third rows, and 30 μm for the second and fourth rows. The optical density of SERPINB9 staining was normalized by subtracting the optical density value of isotype control from the same slide and summarized in **(B)**. N = 4. Data represent mean ± SD. Comparison of means was performed by one-way ANOVA with Tukey's multiple-comparisons test (ns: P > 0.05, * P < 0.05, ** P< 0.01, *** P< 0.001).

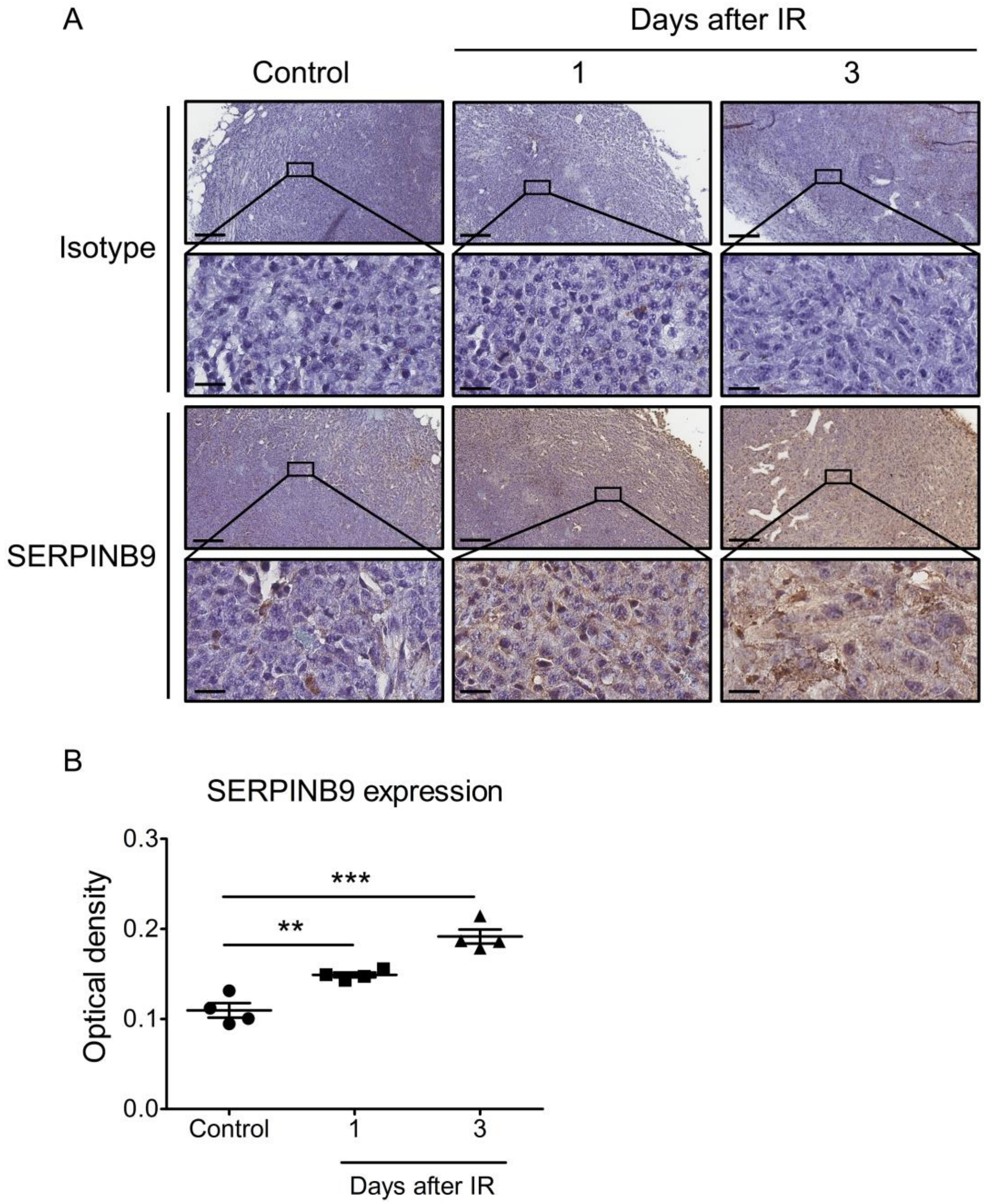

A
Days after IR
Control
1
3
Isotype
SERPINB9
B
SERPINB9 expression
0.3
0.2
0.1
0.0
Optical density
***
**
Control
1
3
Days after IR

Figure S9. Induction of SERPINB9 expression by IR in B16F10 tumor sections. WT B16F10 tumors in C57BL/6 mice were collected at day 0 (control), 1 and 3 following 15 Gy IR. Expression of SERPINB9 in these tumors was assessed by immunohistochemical staining. Representative images are shown in **(A)**. Tissue sections were staining with either rabbit isotype control Ab (the first and second rows) or rabbit anti-SERPINB9 Ab (the third and fourth rows). The staining was developed by Horseradish peroxidase/ 3,3'-Diaminobenzidine (brown) with hematoxylin counterstain (purple). The scale bar is 200 μm for the first and third rows, and 30 μm for the second and fourth rows. The optical density of SERPINB9 staining was normalized by subtracting the optical density value of isotype control from the same slide and summarized in **(B)**. N = 4. Comparison of means was performed by one-way ANOVA with Tukey's multiple-comparisons test (ns: $P > 0.05$, * $P < 0.05$, ** $P < 0.01$, *** $P < 0.001$).

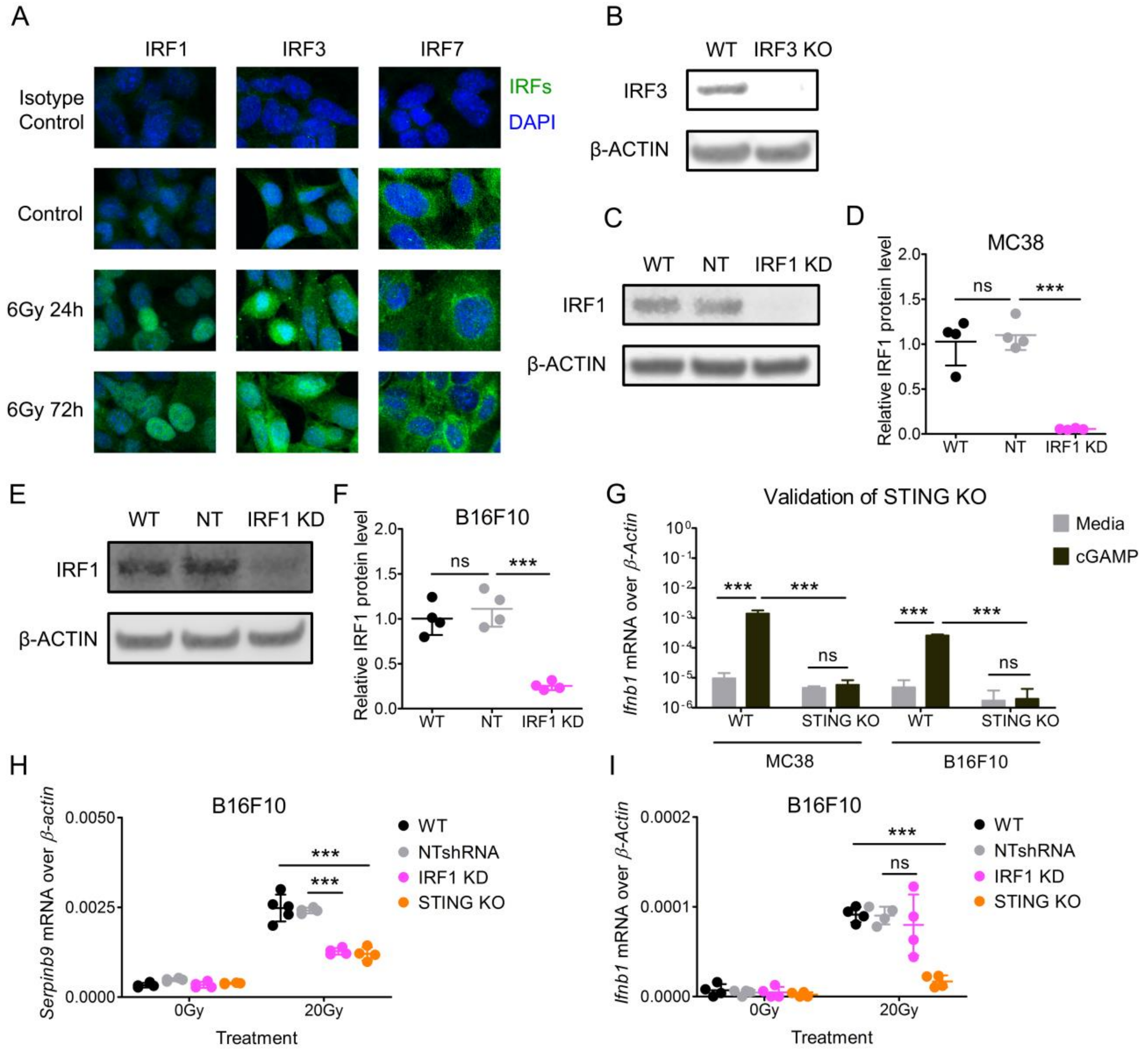
A
IRF1
IRF3
IRF7
IRFs
DAPI
Isotype Control
Control
6Gy 24h
6Gy 72h
B
WT
IRF3 KO
IRF3
β-ACTIN
C
WT
NT
IRF1 KD
IRF1
β-ACTIN
D
MC38
Relative IRF1 protein level
ns
***
WT
NT
IRF1 KD
E
WT
NT
IRF1 KD
IRF1
β-ACTIN
F
B16F10
Relative IRF1 protein level
ns
***
WT
NT
IRF1 KD
G
Validation of STING KO
Ifnb1 mRNA over β-Actin
Media
cGAMP
***
***
ns
***
***
ns
WT
STING KO
WT
STING KO
MC38
B16F10
H
B16F10
Serpinb9 mRNA over β-actin
WT
NTshRNA
IRF1 KD
STING KO
***
***
0Gy
20Gy
Treatment
I
B16F10
Ifnb1 mRNA over β-Actin
WT
NTshRNA
IRF1 KD
STING KO
***
ns
0Gy
20Gy
Treatment

Figure S10 IR augmented expression of Serpinb9 in cancer cells via STING-mediated type I IFN induction as well as IFN signaling-independent mechanisms. **(A)**: Distribution of IRFs (IRF1, IRF3 and IRF7) in MC38 cells 24 hours following 0 Gy (second row), 24 or 72 hours following 6 Gy IR (third and fourth row). Irradiated cells stained with rabbit isotype control Ab are shown in the first row. IRFs are in green and DAPI in blue. The KO of IRF3 in MC38 cells was validated in **(B)**. The knockdown efficacy of IRF1 in MC38 cells and B16F10 cells (WT, WT cells transfected with non-targeting shRNA lentivirus (NTshRNA), IRF1 knockdown (KD)) was assessed by Western blot **(C-F)**. N = 4 in both **(D)** and **(F)**. B-ACTIN was used as the loading control. The KO of STING in MC38 and B16F10 cells was validated in **(G)**. WT and STING KO MC38 and B16F10 cells were treated with control media or cGAMP (delivered via Lipofectamine 3000) for 6 hours in tissue culture. Following treatment, *Ifnb1* expression in these cells was evaluated by RT-qPCR. N = 4. **(H)** and **(I)**: *Serpinb9* and *Ifnb1* expression in B16F10 cells (WT, NTshRNA, IRF1 KD and STING KO) at 72 hours after 20Gy. N = 4. All mRNA expression levels were normalized to *β-actin*. Data represent mean ± SD. Comparison of means was performed by one-way ANOVA with Tukey's multiple-comparisons test (ns: $P > 0.05$, * $P < 0.05$, ** $P < 0.01$, *** $P < 0.001$).

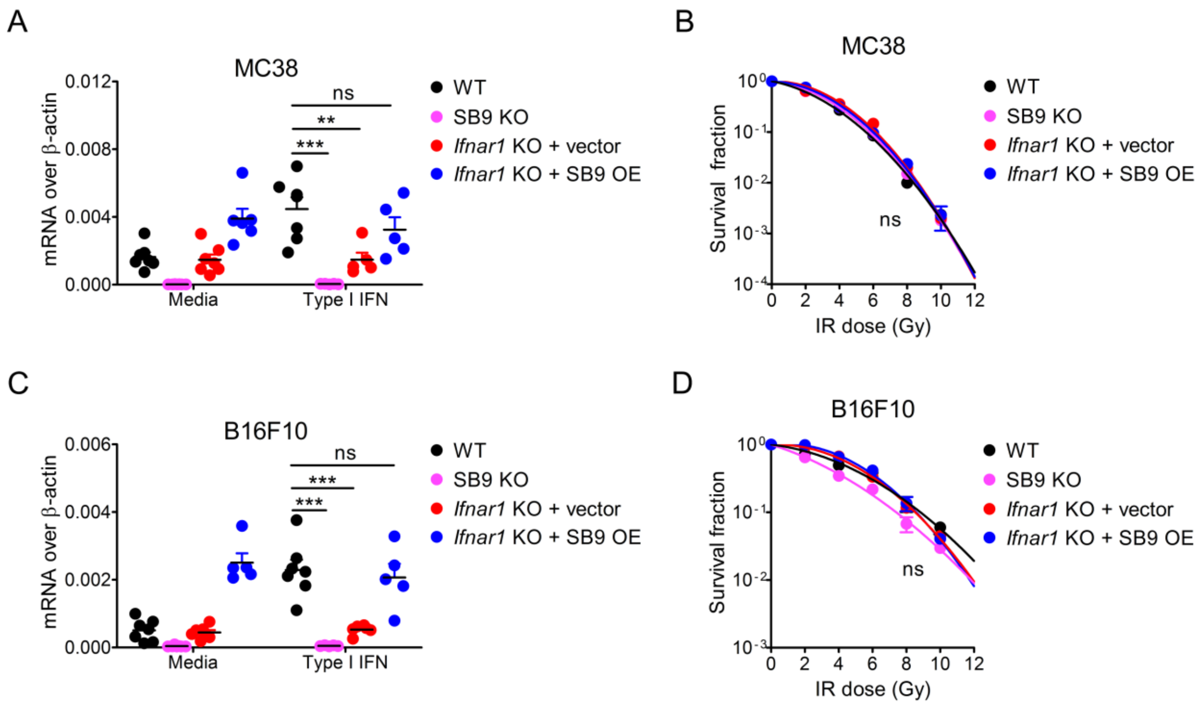

Figure S11 MC38 and B16F10 cells with different phenotypes are indistinguishable in radiosensitivity. MC38 and B16F10 cells (4 phenotypes for each cell line: WT, *Serpinb9* KO (SB9 KO), *Ifnar1* KO + vector, *Ifnar1* KO + *Serpinb9* overexpression (SB9 OE)) were stimulated with control media or type I IFN (50 U/ml) for 4 hours. The expression of *Serpinb9* was assessed with data summarized in **(A)** and **(C)**. N = 5-7. Radiosensitivity of these cells was evaluated by clonogeneic assay and summarized in **(B)** and **(D)**. N = 3. Data represent the mean ± SD. Comparison of means was performed by one-way ANOVA with Tukey's multiple-comparisons test (ns: P > 0.05, * P < 0.05, ** P< 0.01, *** P< 0.001).

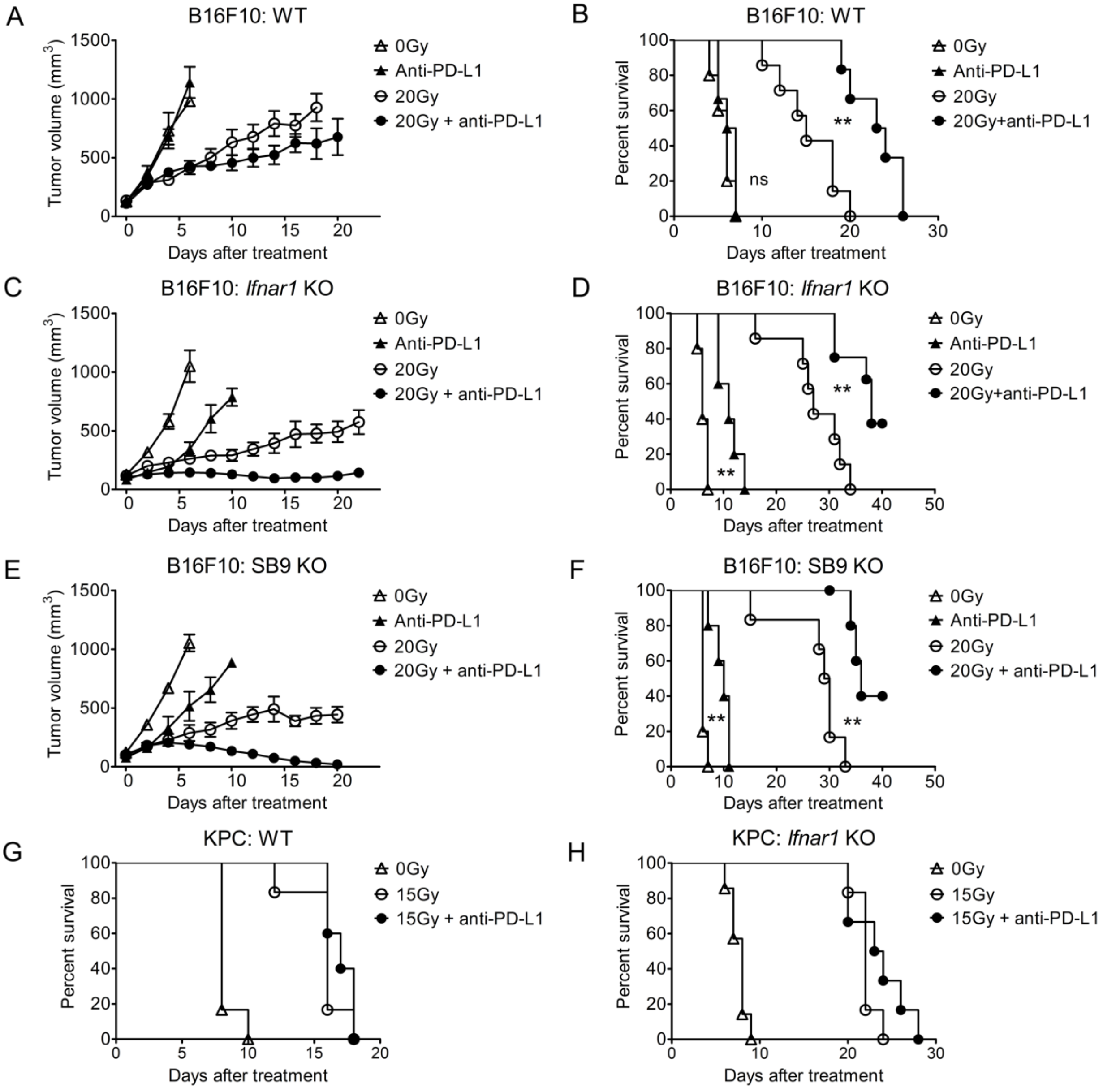

A
B16F10: WT
Tumor volume (mm3)
Days after treatment
0Gy
Anti-PD-L1
20Gy
20Gy + anti-PD-L1
B
B16F10: WT
Percent survival
Days after treatment
ns
**
0Gy
Anti-PD-L1
20Gy
20Gy+anti-PD-L1
C
B16F10: Ifnar1 KO
Tumor volume (mm3)
Days after treatment
0Gy
Anti-PD-L1
20Gy
20Gy + anti-PD-L1
D
B16F10: Ifnar1 KO
Percent survival
Days after treatment
**
**
0Gy
Anti-PD-L1
20Gy
20Gy+anti-PD-L1
E
B16F10: SB9 KO
Tumor volume (mm3)
Days after treatment
0Gy
Anti-PD-L1
20Gy
20Gy + anti-PD-L1
F
B16F10: SB9 KO
Percent survival
Days after treatment
**
**
0Gy
Anti-PD-L1
20Gy
20Gy + anti-PD-L1
G
KPC: WT
Percent survival
Days after treatment
0Gy
15Gy
15Gy + anti-PD-L1
H
KPC: Ifnar1 KO
Percent survival
Days after treatment
0Gy
15Gy
15Gy + anti-PD-L1

Figure S12. ***Ifnar1*** **KO or** ***Serpinb9*** **KO B16F10 or KPC tumors exhibited greater levels of response to anti-PD-L1.** C57BL/6 mice bearing subcutaneous B16F10 (WT, *Ifnar1* KO and *Serpinb9* (SB9) KO or KPC (WT and *Ifnar1* KO) were subjected to the following treatments: (1) 0 Gy IR; (2) anti-PD-L1; (3) IR (20Gy for B16F10 and 15Gy for KPC) on day 0; (4) IR plus anti-PD-L1 Ab. Anti-PD-L1 was given on days -1, 3, 7 and 11. Following IR, tumor volumes were measured every other day and summarized in **(A)**, **(C)** and **(E)**. Kaplan-Meier survival curves of mice were plotted in **(B)**, **(D)** and **(F-H)**. N = 5-8. Data represent mean ± SEM. Comparison of two means was performed by the Mann-Whitney U test. Survival comparisons between groups were performed using the log-rank's test (ns: P > 0.05, * P < 0.05, ** P< 0.01, *** P< 0.001).

Table S1. List of antibodies used in this study

Antibodies	Sources	Cat#	Dilutions	Application
Rat anti-mouse CD16/CD32	BD Bioscience	553141	1:50	Fc block
FITC anti-mouse CD8a (clone 53-6.7)	eBioscience	11-0081-81	1:160	FC
FITC anti-mouse CD31 (clone 390)	eBioscience	11-0311-82	1:160	FC
FITC anti-mouse CD140a (clone APA5)	eBioscience	11-1401-80	1:80	FC
Alexa Fluor 488 anti-mouse CEACAM1a (clone MAb-CC1)	BioLegend	134525	1:200	FC
PerCP-Cy5.5 anti-mouse Galectin-9 (clone RG9-35)	BioLegend	136111	1:160	FC
PE anti-Ki-67 (clone SolA15)	eBioscience	12-5698-82	1:660	FC
PE anti-mouse CD86 (clone B7-2)	eBioscience	12-0862-81	1:160	FC
PE anti-mouse HVEM (clone HMHV-1B18)	BioLegend	136303	1:40	FC
PE-Cy7 anti-mouse CD45 (clone 30-F11)	eBioscience	25-0451-81	1:160	FC
PE-Cy7 anti-mouse Granzyme B (clone NGZB)	eBioscience	25-8898-82	1:330	FC
PE-Cy7 anti-mouse CD3ε (clone 145-2C11)	eBioscience	25-0031-81	1:40	FC
APC anti-mouse Tim-3 (clone RMT3-23)	BioLegend	119705	1:160	FC
APC anti-mouse IFNγ (clone XMG1.2)	eBioscience	17-7311-81	1:160	FC
APC anti-mouse CD31 (clone 390)	eBioscience	17-0311-082	1:160	FC
APC anti-mouse CD140a (clone APA5)	eBioscience	17-1401-81	1:80	FC
APC anti-mouse NKp46 (clone 29A1.4)	BioLegend	137607	1:160	FC
Alexa Fluor 700 anti-mouse CD45 (clone 30-F11)	eBioscience	56-0451-82	1:160	FC
eFluor 780 Fixable Viability Dye	eBioscience	65-0865-14	1:1000	FC
BV421 anti-mouse PD-L1 (clone 10F.9G2)	BioLegend	124315	1:80	FC
BV 421 anti-mouse PD-1 (clone 29F.1A12)	BioLegend	135221	1:80	FC
BV510 anti-mouse CD3ε (clone 145-2C11)	BioLegend	100353	1:40	FC

BV605 anti-mouse CD3ε (clone 145-2C11)	BioLegend	100351	1:40	FC
BV785 anti-mouse CD8a (clone 53-6.7)	BioLegend	100749	1:160	FC
Alexa Fluor 488 conjugated Goat anti-Rabbit IgG (H+L) Secondary Antibody	Thermo Fisher Scientific	A-11034	1:500	IF
IRF-1 (clone D5E4) XP rabbit monoclonal antibody	Cell Signaling Technology	8478	1:200/1: 1000	IF/WB
IRF-3 (clone FL-425) rabbit polyclonal antibody	Santa Cruz Biotechnology	sc-9082	1:100	IF
IRF7 (clone EPR4718) rabbit monoclonal antibody	Abcam	ab109255	1:500	IF
Rabbit IgG Control	R & D	AB-105-C	Accordingly	IF/IHC
SerpinB9 antibody	GeneTex	GTX54693	1:500	IHC
β-Actin (clone 8H10D10) Mouse mAb	Cell Signaling Technology	3700	1:5000	WB
IRDye 800CW Goat Anti-Rabbit IgG (H+L)	Li-cor	926-32211	1:10000	WB
IRDye 680RD Goat Anti-Mouse IgG (H+L)	Li-cor	926-68070	1:10000	WB
InVivoMAb rat anti-mouse CD8α (clone 2.43)	Bio X cell	BE0061	NA	Cells in vivo depletion
InVivoMAb rat IgG2b isotype control (clone LTF-2)	Bio X cell	BE0090	NA	Isotype control
InVivoMAb mouse anti-mouse NK1.1 (clone PK136)	Bio X cell	BE0036	NA	Cells in vivo depletion
InVivoMAb mouse IgG2a isotype control (clone C1.18.4)	Bio X cell	BE0085	NA	Isotype control
InVivoMAb rat anti-mouse PD-L1 (clone 10F.9G2)	Bio X cell	BE0101	NA	In vivo blockade

Cat#: catalogue number; NA: Not applicable; FC: Flow cytometry; IF: Immunofluorescence staining; IHC: Immunohistochemistry; WB: Western blot.

Table S2. Primers for SBYR Green-based RT-qPCR

Genes	Accessions		Sequences (5' - 3')	Amplicon lengths
Serpinb9	NM_009256	F	TCAGGTGGCTCCGTCGATT	107
		R	GGCATGTCCATTGTGTACTCTT	
Serpinb9b	NM_011452	F	CCTGACTGCTCACAAGCC	207
		R	AGTAAAACCCATAGCACT	
Bcl2	NM_009741	F	ATGCCTTTGTGGAACTATATGGC	120
		R	GGTATGCACCCAGAGTGATGC	
Bcl2l1	NM_001191	F	AACATCCCAGCTTCACATAACCCC	93
		R	GCGACCCCAGTTTACTCCATCC	
Cd274	NM_021893	F	CAAGTGAGAATGCTAGATGTG	176
		R	TCCATCTTGAGTCTTTGGAC	
Lgals9	NM_010708	F	ATGCCCTTTGAGCTTTGCTTC	218
		R	AACTGGACTGGCTGAGAGAAC	
Pvr	NM_027514	F	CAACTGGTATGTTGGCCTCA	198
		R	ATTGGTGACTTCGCACACAA	
H2-K1	NM_001001892	F	GCTGGTGAAGCAGAGAGACTCAG	154
		R	GGTGACTTTATCTTCAGGTCTGCT	
Ifnb1	NM_002176	F	CAGCTCCAAGAAAGGACGAAC	137
		R	GGCAGTGTAACTCTTCTGCAT	
β-actin	NM_007393	F	CGGTTCCGATGCCCTGAGGCTCTT	99
		R	CGTCACACTTCATGATGGAATTGA	

F: forward

R: Reverse

Table S3. Primers/probes for TaqMan-based RT-qPCR

Genes	Accessions	Primers/probes
Ifit1	NM_008331	Mm00515153_m1
Ifi44	NM_133871	Mm00505670_m1
β-actin	NM_007393	Mm02619580_g1

Table S4. CRISPR gRNA sequences

Genes	Targeted exons	gRNA Pair	Sequences (5'-3')
Ifnar1	4	1	CCACATGTTCCCGTCTTGTC
		2	TCAGTTACACCATACGAATC
Serpinb9	1	1	CAGAGCTGCCTGTACAAGGC
		2	AGCATCAAGGCTGCAAACGC
Tmem173	3	1	GTGGATCCTTTGCCACCCAA
(*Sting*)		2	CACCTAGCCTCGCACGAACT
Irf3	1	1	CAGCTAAGCCTCCGAGCGCT
		2	CGAGTCTCAGAACTACTGTT

PRAD	THCA	BRCA	PAAD	LUAD	KIRC	LGG	COAD	BLCA	KIRP	LUSC	LIHC	STAD	HNSC	SKCM	CESC	GENE NAME
0.83	0.73	0.73	0.61	0.6	0.52	0.63	0.55	0.53	0.56	0.56	0.59	0.5	0.51	0.53	0.38	*CSF2RB*
0.76	0.66	0.69	0.63	0.58	0.52	0.67	0.53	0.59	0.49	0.55	0.52	0.5	0.43	0.52	0.35	*CD86*
0.76	0.67	0.68	0.65	0.47	0.48	0.65	0.52	0.4	0.51	0.53	0.49	0.48	0.54	0.48	0.38	*CD69*
0.84	0.59	0.66	0.58	0.59	0.61	0.54	0.57	0.57	0.28	0.47	0.28	0.39	0.35	0.49	0.34	*GBP1*
0.73	0.65	0.64	0.56	0.53	0.44	0.5	0.53	0.49	0.4	0.53	0.41	0.25	0.26	0.46	0.46	*BIRC3*
0.79	0.66	0.61	0.54	0.61	0.54	0.33	0.38	0.57	0.54	0.26	0.54	0.42	0.45	-0.06	0.2	*RUNX3*
0.5	0.61	0.54	0.47	0.51	0.53	0.37	0.55	0.54	0.21	0.46	0.41	0.32	0.37	0.41	0.39	*STAT1*
0.69	0.59	0.53	0.52	0.52	0.34	0.44	0.41	0.48	0.47	0.53	-0.01	0.41	0.36	0.49	0.37	*SERPING1*
0.88	0.65	0.68	0.64	0.4	0.66	0.42	0.46	0.28	0.49	0.05	0.56	0.46	0.2	0.05	0.14	*IFI16*
0.56	0.58	0.53	0.35	0.51	0.47	0.35	0.49	0.42	0.45	0.54	0.33	0.22	0.34	0.5	0.31	*IRF1*
0.79	0.56	0.67	0.39	0.32	0.37	0.56	0.05	0.14	0.39	0.48	0.5	0.15	0.51	0.45	0.29	*CTSS*
0.43	0.57	0.57	0.48	0.51	0.37	0.37	0.48	0.58	0.21	0.48	0.34	0.31	0.36	0.49	0.35	*CXCL10*
0.56	0.51	0.48	0.56	0.45	0.37	0.52	0.47	0.47	0.4	0.51	0.31	0.31	0.25	0.42	0.17	*SLC15A3*
0.62	0.09	0.6	0.53	0.44	0.38	0.25	0.53	0.25	0.41	0.52	0.42	0.45	0.44	0.43	0.33	*RASGRP3*
0.63	0.51	0.52	0.52	0.41	0.53	0.44	0.49	0.22	0.36	0.41	0.18	0.24	0.22	0.4	0.3	*XAF1*
0.52	0.41	0.47	0.52	0.41	0.43	0.17	0.45	0.56	0.28	0.46	0.38	0.37	0.35	0.43	0.23	*SIGLEC1*
0.74	0.54	0.47	0.34	0.46	0.6	0.37	0.47	0.36	0.3	0.25	0.47	0.17	0.19	0.25	0.26	*SAMD9*
0.59	0.38	0.65	0.61	0.14	0.42	0.35	0.42	0.36	0.27	0.19	0.37	0.39	0.35	0.48	0.12	*LAMP3*
0.39	0.46	0.34	0.55	0.31	0.5	0.56	0.34	0.44	0.39	0.5	0.26	0.33	0.41	0.32	0.26	*RNASE2*
0.56	0.56	0.54	0.39	0.14	0.55	0.55	0.41	0.02	0.4	0.2	0.38	0.26	0.22	0.25	0.16	*MX2*
0.32	0.46	0.44	0.47	0.41	0.57	0.46	0.44	0.21	0.34	0.33	0.3	0.22	0.23	0.29	0.27	*OAS2*
0.68	0.52	0.5	0.42	0.39	0.25	0.51	0.18	0.42	0.24	0.26	0.46	0.09	0.25	0.05	0.29	*PLSCR1*
0.54	0.35	0.47	0.5	0.3	0.4	0.48	0.35	0.34	0.22	0.38	0.28	0.27	0.2	0.26	0.23	*IFI44L*
0.35	0.3	0.4	0.5	0.38	0.2	0.38	0.45	0.55	0.27	0.5	0.18	0.3	0.29	0.37	0.27	*IFI30*
0.58	0.47	0.45	0.4	0.35	0.36	0.29	0.49	0.41	-0.02	0.41	0.15	0.29	0.24	0.28	0.29	*IFIT3*
0.49	0.36	0.45	0.51	0.33	0.4	0.32	0.43	0.41	0.2	0.38	0.2	0.29	0.24	0.35	0.18	*RSAD2*
0.62	0.59	0.49	0.42	0.32	0.41	0.54	0.05	0.01	0.45	0.27	0.42	-0.08	0.31	0.44	0.04	*LGALS9*
0.28	0.5	0.45	0.36	0.44	0.31	0.44	0.34	0.47	0.02	0.44	0.23	0.21	0.27	0.29	0.28	*IFIH1*
0.56	0.45	0.42	0.5	0.34	0.39	0.25	0.47	0.41	0.05	0.36	0.15	0.32	0.25	0.27	0.25	*IFIT2*
0.37	0.51	0.51	0.55	0.31	0.41	0.23	0.52	0.24	0.34	0.3	0.123	0.38	0.23	0.17	0.27	*STAT2*
0.18	0.54	0.37	0.53	0.29	0.44	0.5	0.46	0.21	0.35	0.35	0.21	0.31	0.34	0.25	0.18	*IL1R1*
0.46	0.43	0.45	0.53	0.3	0.51	0.43	0.34	0.31	0.33	0.35	0.21	0.18	0.11	0.2	0.15	*IFI44*
0.32	0.53	0.32	0.58	0.34	0.41	0.32	0.36	0.43	0.21	0.44	0.17	0.31	0.15	0.33	0.26	*SP110*
0.48	0.54	0.35	0.31	0.4	0.37	0.35	0.41	0.38	0.07	0.36	0.18	0.11	0.24	0.28	0.24	*DDX60*
0.43	0.54	0.34	0.38	0.34	0.53	0.2	0.44	0.22	0.27	0.3	0.26	0.12	0.32	0.24	0.27	*OAS3*
0.55	0.31	0.33	0.4	0.29	0.33	0.42	0.6	0.33	0.23	0.29	0.15	0.22	0.2	0.33	0.14	*MX1*
0.5	0.17	0.51	0.32	0.33	0.12	0.27	0.47	0.52	0.27	0	0.38	0.3	0.05	0.43	0.2	*SOCS1*
0.43	0.47	0.33	0.36	0.32	0.3	0.27	0.43	0.43	0.04	0.28	0.17	0.32	0.17	0.3	0.24	*DDX58*
0.53	0.51	0.21	0.51	0.18	0.36	0.33	0.06	0.48	0.22	0.35	0.2	0.28	0.13	0.33	0.38	*IFITM1*
0.45	0.46	0.36	0.35	0.34	0.37	0.39	0.38	0.22	0.08	0.37	0.01	0.07	0.14	0.33	0.23	*OASL*
0.66	0.3	0.372	0.2	0.22	0.31	0.56	0.21	0.1	0.16	0.36	0.26	-0.01	0.42	0.12	0.11	*TLR3*
0.46	0.53	0.33	0.35	0.14	0.23	0.41	0.18	0.49	0.42	0.28	0.16	0.05	0.17	0.18	0.35	*CXCL2*
0.54	0.47	0.37	0.28	0.25	0.2	0.01	0.44	0.34	0.24	0.31	0.13	0.33	0.29	0.15	0.25	*IFIT5*
0.3	0.35	0.45	0.33	0.35	0.12	0.51	0.21	0.47	0.08	0.31	0.12	0.3	0.18	0.18	0.03	*HERC5*
0.04	0.41	0.32	0.22	0.33	0.32	0.39	0.38	0.32	0.3	0.35	0.16	0.17	-0.01	0.44	0.27	*PARP12*
0.47	0.46	0.34	0.36	0.21	0.05	0.29	0.3	0.44	0.42	0.18	0.45	0.09	0.15	-0.03	0.29	*CXCL1*
0.36	0.33	0.37	0.42	0.29	0.36	0.3	0.15	0.34	0.35	0.16	0.38	0.02	0.07	0.15	0.25	*HBEGF*
0.2	0.45	0.39	0.19	0.24	0.16	0.41	0.35	0.32	-0.04	0.24	0.2	0.17	0.19	0.26	0.16	*HERC6*
0.3	0.34	0.34	0.37	0.19	0.36	0.25	0.36	0.44	0.04	0.09	0.26	0.16	0.32	0.1	0.21	*EIF2AK2*
0.52	0.46	0.05	0.06	0.18	0.32	0.69	0.15	0.43	0.39	0.18	0.06	-0.02	0.26	0.22	0.17	*IL13RA1*
0.32	0.43	0.26	0.14	0.31	0.2	0.24	0.22	0.29	0.23	0.33	0.2	0.12	0.09	0.3	0.19	*HLA-G*
0.4	0.33	-0.02	0.4	0.13	0.22	0.43	0.16	0.39	0.34	0.43	0.13	0.23	0.22	0.24	0.39	*IFITM2*
0.46	0.42	0.37	0.27	0.35	0.09	0.29	0.22	0.09	0.25	0.26	-0.04	0.08	0.15	0.13	0.17	*RTP4*
0.08	0.29	0.26	0.25	0.01	0.37	0.36	0.46	0.45	0.24	0.32	0.11	0.17	0.12	0.19	0.19	*ISG20*
0.16	0.41	0.18	0.33	0.35	0.27	0.27	0.2	0.27	0.28	0.09	0.07	0.13	0.21	0.48	0	*TNFSF10*
0.45	0.43	0.14	0.41	0.15	0.03	0.46	0.39	0.16	0.18	0.3	0.07	0.17	0.03	0.23	0.12	*BST2*
0.26	0.44	0.34	0.32	0.23	0.24	0.51	0.16	0.12	0.02	0.12	0.14	0.08	0.1	-0.01	0.14	*SPATS2L*
0.25	0.52	0.02	0.37	0.19	0.46	0.19	0.35	0.15	0.26	0.26	0.14	0.14	0.12	0.28	0.14	*IRF9*
0.33	0.46	0.26	0.26	0.22	0.23	0.41	0.15	-0.01	0.1	0.19	0.07	0	0.13	0.29	0.16	*OAS1*
0.24	0.29	0.37	0.37	0.15	0.24	0.2	0.13	0.22	0.26	0.17	0.1	0.2	0.11	0.21	0.04	*IRGM*
0.43	0.26	0.25	0.32	0.18	0.04	0.22	0.33	0.39	-0.04	0.27	-0.03	0.24	0.13	0.15	0.18	*IFIT1*
0.39	0.15	0.14	0.43	0.41	0.26	0.41	0.08	0.36	0.27	0.04	0.36	0.16	0.05	-0.13	0.18	*CD44*
0.52	0.43	-0.17	0.23	0.14	0.34	0.52	0.12	0.32	0.39	0.03	0.42	-0.03	-0.05	0.07	0.22	*IER3*
0.17	0.22	0.13	0.27	0.26	0.14	0.15	0.22	0.31	0.03	0.36	-0.04	0.14	0.08	0.25	0.21	*IFI35*
0.09	0.24	0.25	0.1	0.04	0.38	0.09	0.32	0.22	0.3	0.11	0.16	-0.05	0.26	0.11	0.12	*USP18*
0.16	0.41	0.17	0.38	0.03	0.27	0.03	0.21	0.25	0.04	0.28	0.19	0.11	0.03	0.11	0.16	*Ly6E*
0.31	0.19	0.23	0.28	0.17	0.12	0.22	0.13	0.27	0	0.24	0.03	-0.07	-0.03	0.19	0.14	*IFI27*
0.13	0.18	0.16	0.09	0.11	0.04	0.25	0.3	0.34	0.12	0.26	0.05	0.08	0.05	0.1	0.19	*IFI6*
0.48	0.15	0.39	0.02	0.01	0.04	-0.31	0.19	-0.09	0.3	0.3	0.12	-0.13	0.21	0.17	0.25	*BMP2*
-0.15	0.31	0.08	0.28	-0.03	0.15	0.29	0.22	0.14	0.33	0.33	0.03	0.07	0.12	0.17	0.22	*IRF7*
0.06	0.19	0.13	0.34	0.08	0.09	0.23	0.34	0.22	0.04	0.22	0.01	0.17	-0.05	0.21	0.18	*ISG15*
0.4	0.18	0.01	0.1	0.1	0.09	0.35	0.15	0.06	0.18	-0.01	0.22	0.06	0.23	-0.13	0.03	*CIC*
0.43	0.19	0.12	-0.03	0.01	0.14	0.23	0.05	-0.01	0.09	0.09	0.23	-0.22	0.08	-0.01	0.15	*LGALS3BP*
-0.06	0.293	0.03	0.15	0.11	-0.09	0.07	0.22	0.09	-0.12	0.3	-0.09	0.12	0.03	0.38	0.11	*PSME1*
0.56	-0.34	0.16	-0.21	0.08	-0.27	0.36	0.02	-0.08	-0.12	0.01	-0.09	0.08	0.2	0.02	-0.26	*ACACB*
-0.12	0.1	0	0.17	-0.03	0.07	0.32	0.01	-0.09	0.14	0.08	-0.04	0.08	-0.16	0.14	0.13	*IRF3*
0.02	0.06	-0.04	0.11	0.11	0	0.03	-0.1	-0.06	-0.03	-0.02	0.09	-0.17	0.05	-0.02	-0.08	*APOBEC1*

Peason r

- > 0.80
- > 0.60, but ≤ 0.80
- > 0.40, but ≤ 0.60
- > 0.20, but ≤ 0.40
- > 0, but ≤ 0.20
- ≤ 0

Table S5. Expression correlation between *SERPINB9* and IFN signature genes in TCGA data. TCGA gene expression data (RNA-seq V2 RSEM) of 77 IFN signature genes (listed on the first column on the right) and *SERPINB9* in 16 different human cancer types was downloaded via cBioPortal (http://www.cbioportal.org/) and log2 transformed. The case numbers for each cancer type were as follows: Prostate Adenocarcinoma(PRAD), n = 498; Breast Invasive Carcinoma (BRCA), n = 1100; Thyroid Carcinoma (THCA), n = 509; Pancreatic Adenocarcinoma (PAAD), n = 178; Kidney Renal Clear Cell Carcinoma (KIRC), n = 446; Lung Adenocarcinoma (LUAD), n = 517; Kidney Renal Papillary Cell Carcinoma (KIRP), n = 291; Brain Lower Grade Glioma (LGG), n = 530; Lung Squamouse Cell Carcinoma (LUSC), n = 501; Bladder Urothelial Carcinoma (BLCA), n = 408; Colorectal Adenocarcinoma (COAD), n = 382; Liver Heptocellular Carcinoma (LIHC), n = 373; Head and Neck Squamouse Cell Carcinoma (HNSC), n = 522; Stomach Adenocarcinoma (STAD), n = 415; Skin Cutaneous Melanoma (SKCM), n = 472; Cervical Squamouse Cell Carcinoma and Endocervical Adenocarcionoma (CESC), n = 306. The correlation between IFN signature genes and *SERPINB9* was evaluted by Pearson's correlation. Pearson correlation coefficients (r) are indicated. All P values < 0.0001. The genes were ranked according to the mean r value of each row from high to low.
